# Supplementary material for: MycoRed: Betalain pigments enable in vivo real-time visualisation of arbuscular mycorrhizal colonisation
Source: PLoS Biol. 2021 Jul 14;19(7):e3001326. doi: 10.1371/journal.pbio.3001326 (PMC8312983; doi:10.1371/journal.pbio.3001326)

S1 Fig. Expression of arbuscular mycorrhiza symbiosis marker genes *MtSTR* and *MtRAM2* in *Medicago truncatula MtPT4*-p1 composite roots is induced after inoculation with *Rhizophagus irregularis* and shows evidence of functional symbiosis. Expression of *Rhizophagus irregularis* β-tubulin (*RiBTub*) and elongation factor 1-alpha (*RiEF*) is used as a fungal biomass marker for root colonisation. Gene expression was analysed by qRT-PCR 4 weeks post inoculation. Mock roots were inoculated with autoclaved *R. irregularis* inoculum. Error bars represent standard errors of three biological replicates. Statistical p-value (p) was obtained via unpaired two-samples Student's *t* test. Data underlying this figure can be found in S3 Data.

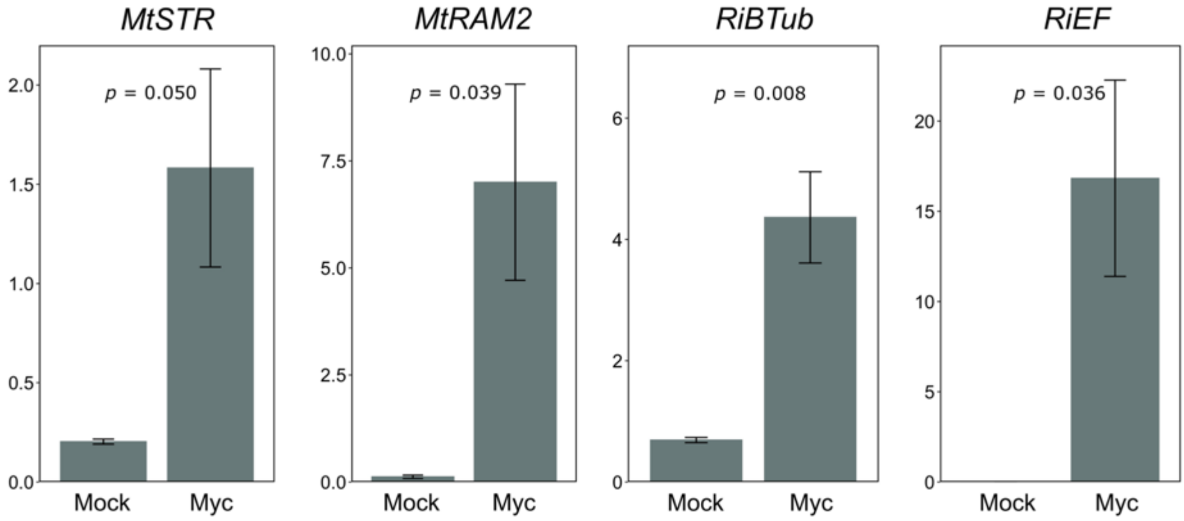

Supplement: S1 Fig — Expression of RiBTub and RiEF is used as a fungal biomass marker for root colonisation. Gene expression was analysed by qRT-PCR 4 wpi. Mock roots were inoculated with autoclaved R. irregularis inoculum. Error bars represent standard errors of 3 biological replicates. Statistical p-value (p) was obtained via unpaired 2-sampled Student t test. Data underlying this figure can be found in S3 Data. AM, arbuscular mycorrhiza; qRT-PCR, quantitative real-time polymerase chain reaction; RiBTub, R. irregularis β-tubulin; RiEF, R. irregularis longation factor 1-alpha; wpi, weeks postinoculation. (PDF) [file pbio.3001326.s001.pdf]
