# Supplementary material for: MycoRed: Betalain pigments enable in vivo real-time visualisation of arbuscular mycorrhizal colonisation
Source: PLoS Biol. 2021 Jul 14;19(7):e3001326. doi: 10.1371/journal.pbio.3001326 (PMC8312983; doi:10.1371/journal.pbio.3001326)

S2 Fig. GUS staining of *Medicago truncatula* roots expressing *MtPT4*::GUS (a,b) and *MtBCP1*::GUS (c,d). GUS staining confirms promoter expression is limited to single cells in typically arbusculated tissue layers (inner cortex). Scale bar, 100 µm.

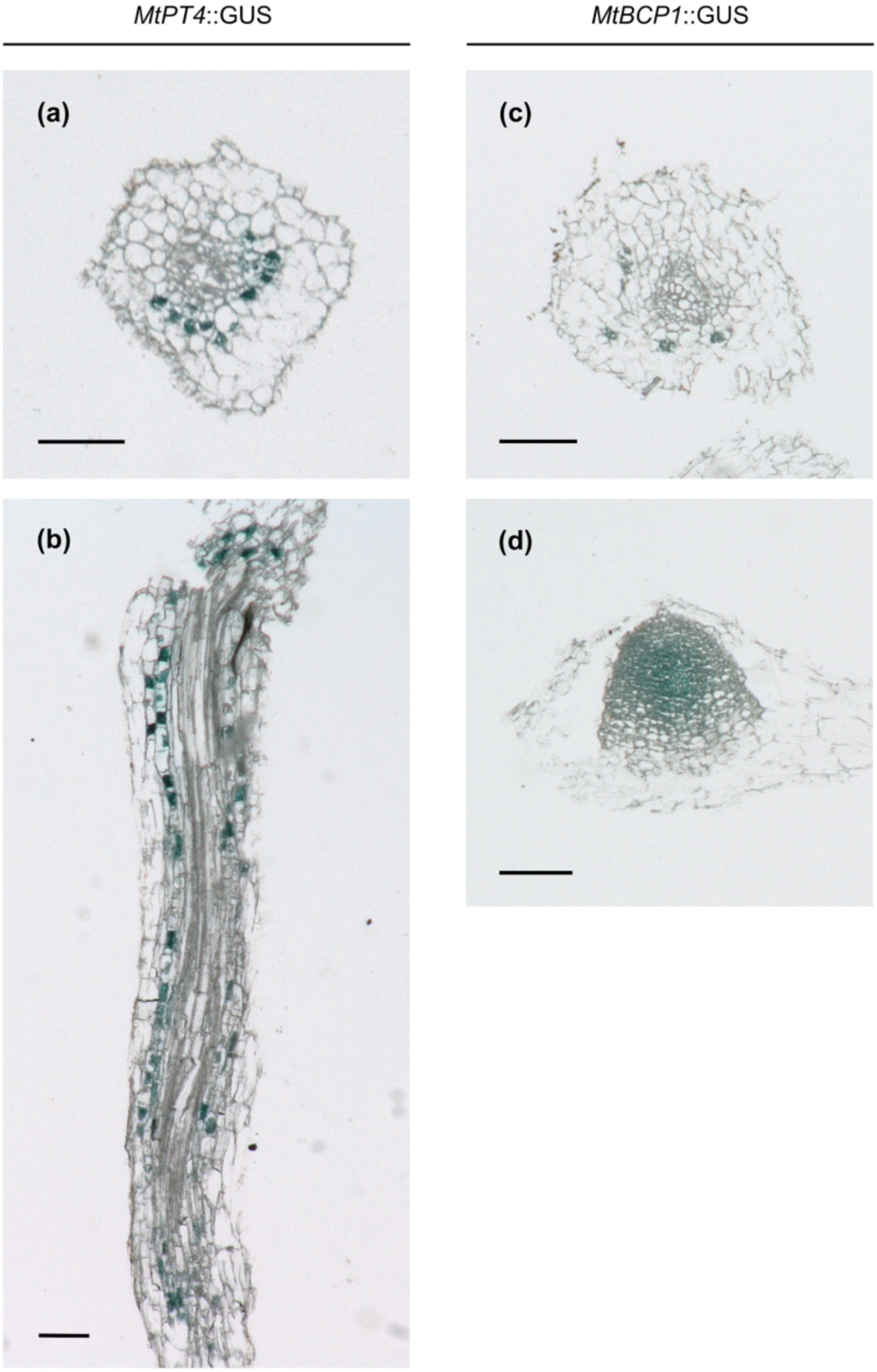

Supplement: S2 Fig — GUS staining of Medicago truncatula roots expressing MtPT4::GUS (a and b) and MtBCP1::GUS (c and d). GUS staining confirms promoter expression is limited to single cells in typically arbusculated tissue layers (inner cortex). Scale bar, 100 μm. (PDF) [file pbio.3001326.s002.pdf]
