# Supplementary material for: MycoRed: Betalain pigments enable in vivo real-time visualisation of arbuscular mycorrhizal colonisation
Source: PLoS Biol. 2021 Jul 14;19(7):e3001326. doi: 10.1371/journal.pbio.3001326 (PMC8312983; doi:10.1371/journal.pbio.3001326)

S3 Fig. Phylogenetic analysis of the PT and BCP1 orthogroups containing the a) *MtPT4 and NbPT5b* homologs and b) *MtBCP1 and NbBCP1b* homologs respectively.

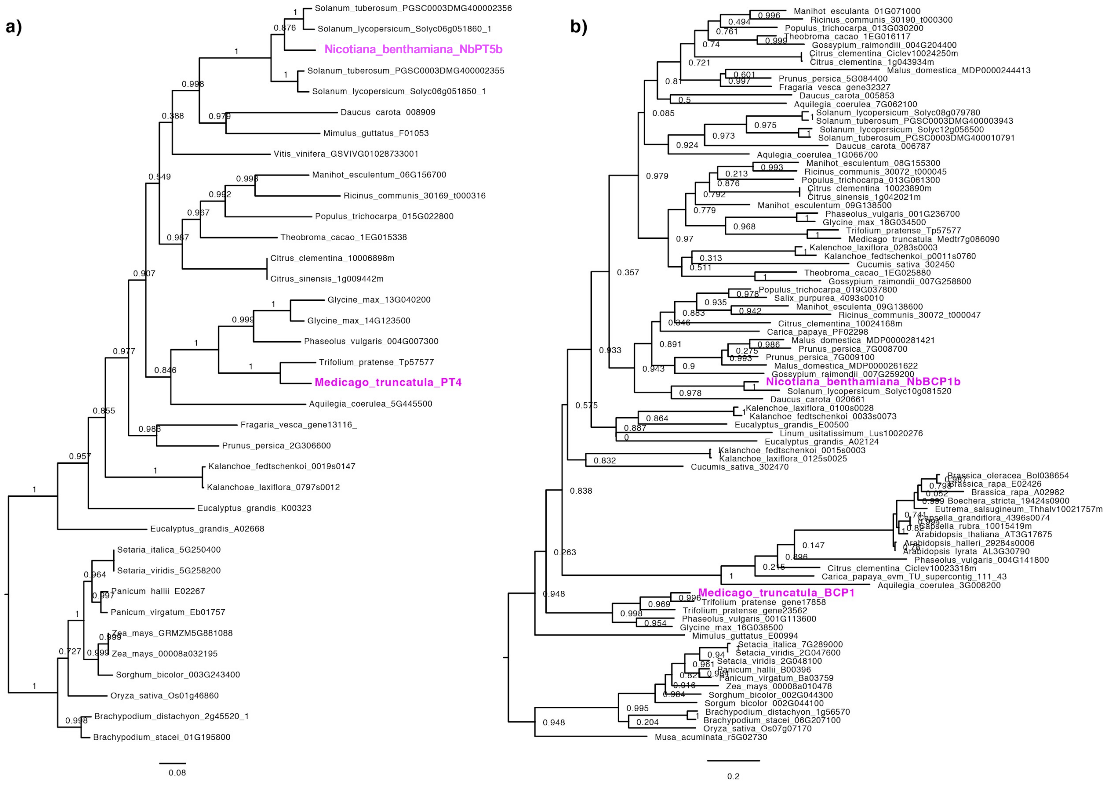

Supplement: S3 Fig — Phylogenetic analysis of the PT and BCP1 orthogroups containing the (a) MtPT4 and NbPT5b homologues and (b) MtBCP1 and NbBCP1b homologues, respectively. (PDF) [file pbio.3001326.s003.pdf]
