# Supplementary material for: MycoRed: Betalain pigments enable in vivo real-time visualisation of arbuscular mycorrhizal colonisation
Source: PLoS Biol. 2021 Jul 14;19(7):e3001326. doi: 10.1371/journal.pbio.3001326 (PMC8312983; doi:10.1371/journal.pbio.3001326)

S4 Fig. 6-week old *Nicotiana benthamiana* T1 plants from *NbPT5b*-p1 (a-d) and *NbBCP1*-p1 (e-h) expressing lines. (a,b,e,f) Plants 4-week after inoculation with *Rhizophagus irregularis*, (c,d,g,h) Plants descending from same lines mock inoculated with autoclaved *R. irregularis* inoculum. Scale bar, 1 cm.

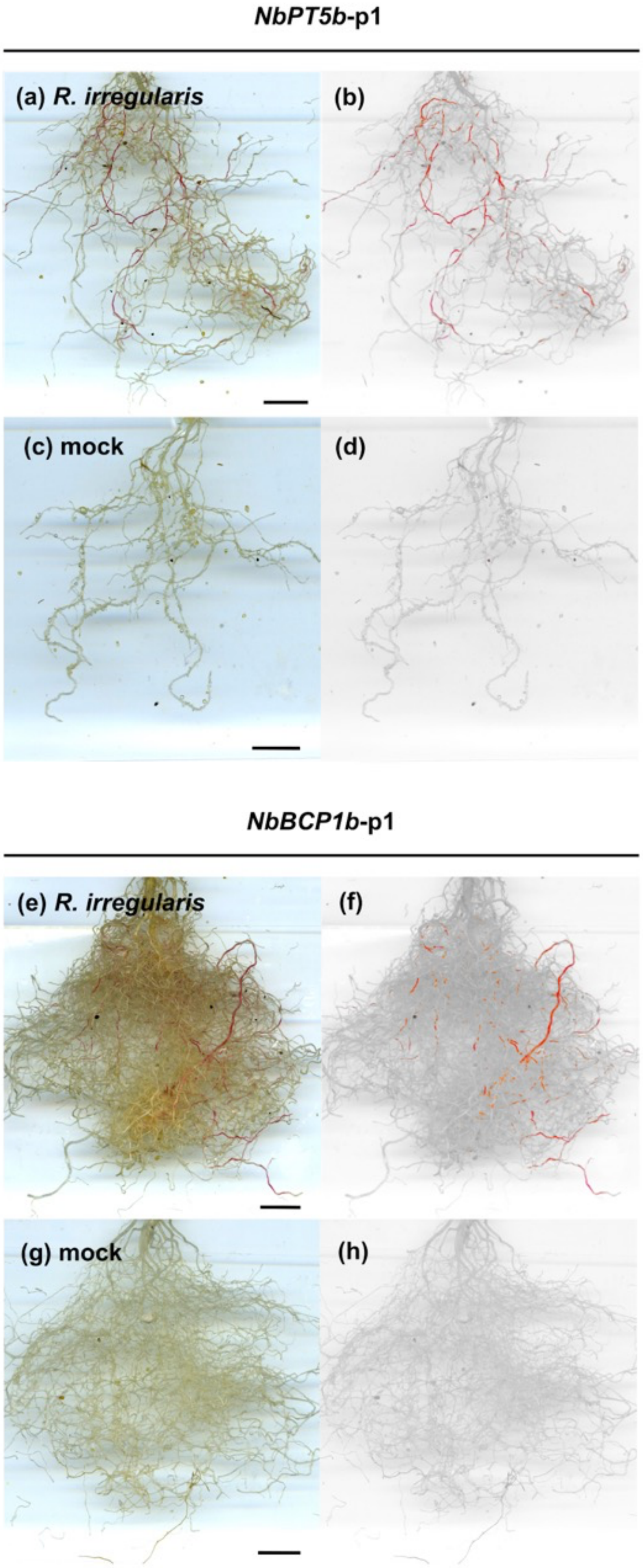

Supplement: S4 Fig — Six-week old Nicotiana benthamiana T1 plants from NbPT5b-p1 (a–d) and NbBCP1-p1 (e–h) expressing lines. (a, b, e, and f) Plants 4 wpi with Rhizophagus irregularis. (c, d, g, and h) Plants descending from same lines mock inoculated with autoclaved R. irregularis inoculum. Scale bar, 1 cm. wpi, weeks after inoculation. (PDF) [file pbio.3001326.s004.pdf]
