# Supplementary material for: MycoRed: Betalain pigments enable in vivo real-time visualisation of arbuscular mycorrhizal colonisation
Source: PLoS Biol. 2021 Jul 14;19(7):e3001326. doi: 10.1371/journal.pbio.3001326 (PMC8312983; doi:10.1371/journal.pbio.3001326)

S5 Fig. Ink staining of *Nicotiana benthamiana* roots expressing *NbPT5b*-p1 (a-d) and *NbBCP1b*-p1 (e-h) after inoculation with *Rhizophagus irregularis*. (a,b,e,f) Root fragments that displayed betalain colouration before ink staining are represented by a red '+' sign. (c,d,g,h) Root fragments that displayed no colouration before ink staining are represented by a grey '-' sign. (b,d,f,h) are amplified images of the area delimited by the dashed squares. Scale bar, 1 mm.

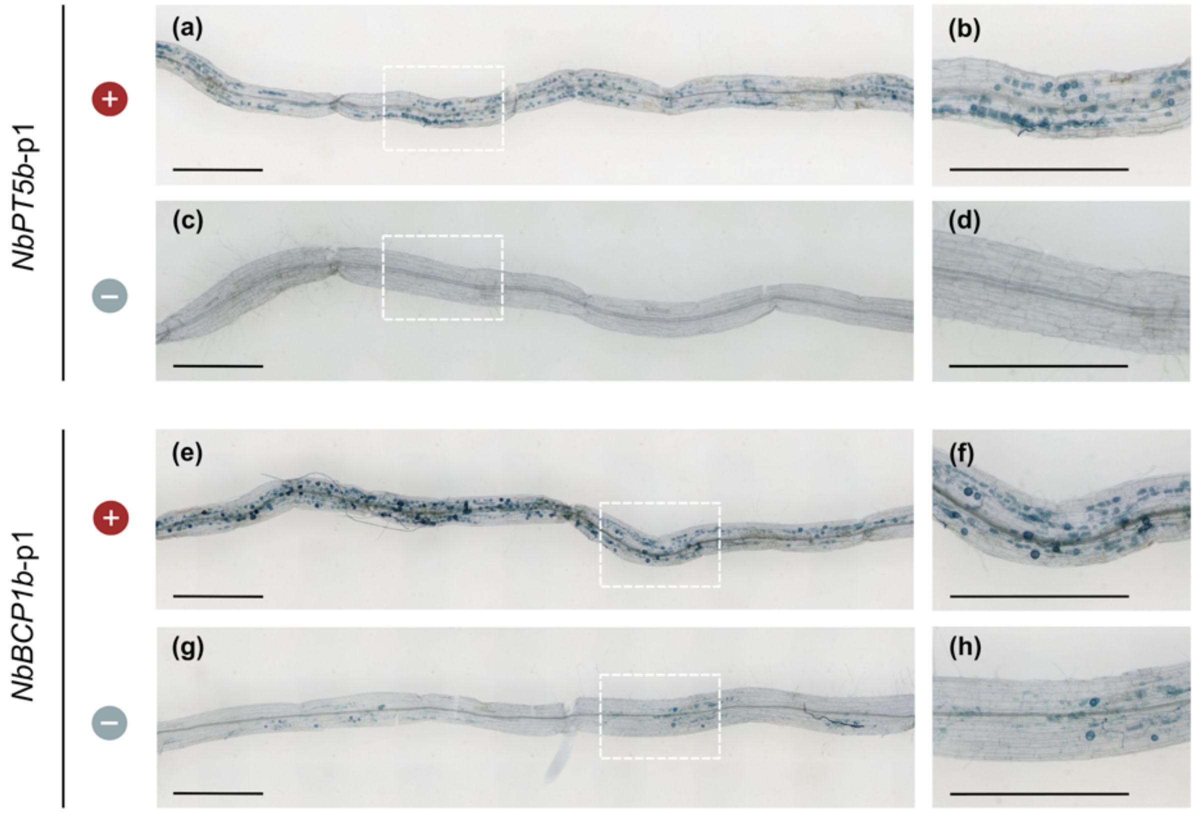

Supplement: S5 Fig — Ink staining of Nicotiana benthamiana roots expressing NbPT5b-p1 (a–d) and NbBCP1b-p1 (e–h) after inoculation with Rhizophagus irregularis. (a, b, e, and f) Root fragments that displayed betalain colouration before ink staining are represented by a red “+” sign. (c, d, g, and h) Root fragments that displayed no colouration before ink staining are represented by a grey “−”sign. (b, d, f, and h) are amplified images of the area delimited by the dashed squares. Scale bar, 1 mm. (PDF) [file pbio.3001326.s005.pdf]
