# Supplementary material for: MycoRed: Betalain pigments enable in vivo real-time visualisation of arbuscular mycorrhizal colonisation
Source: PLoS Biol. 2021 Jul 14;19(7):e3001326. doi: 10.1371/journal.pbio.3001326 (PMC8312983; doi:10.1371/journal.pbio.3001326)

S6 Fig. Expression of *NbPT5b*-p1 in *Nicotiana benthamiana* stable transformants leads to developmental defects. Figure shows examples of T0 plants which varied in the severity of observable developmental defects and were phenotypically classified as severe (S; a,d,e), moderate (M; b,f,g) and light (L; c). Only plants with a light phenotype were able to set seeds, although not abundantly. Defects affected overall plant size, and leaf and flower morphology. Severely affected plants displayed thicker leaves with strong deformation commonly showing a bifurcated shape (d) and flowers lacking reproductive organs (e). Moderately affected plants displayed altered leaf elongation with curvy edges (f), and flowers with a shorter (or lacking) perianth (g). Light phenotypes included slight leaf deformation, and flowers with shorter perianth but typical overall plant growth (c). (h) Semiquantitative RT-PCR analysis of betalain biosynthetic transgene expression in leaves of *N. benthamiana* T0 stable *NbPT5b*-p1 transformants. Line 18 displayed light developmental defects and was able to set seed, line 6 displayed moderate developmental defects, and lines 1 and 15 displayed severe developmental defects. NbEF1α is used as housekeeping control. Positive control (+) is *NbPT5b*-p1 plasmid DNA. Negative control (-) is water. *-RT* stands for minus reverse transcriptase control generated from line 18. Original gel images can be found in S4 Data.

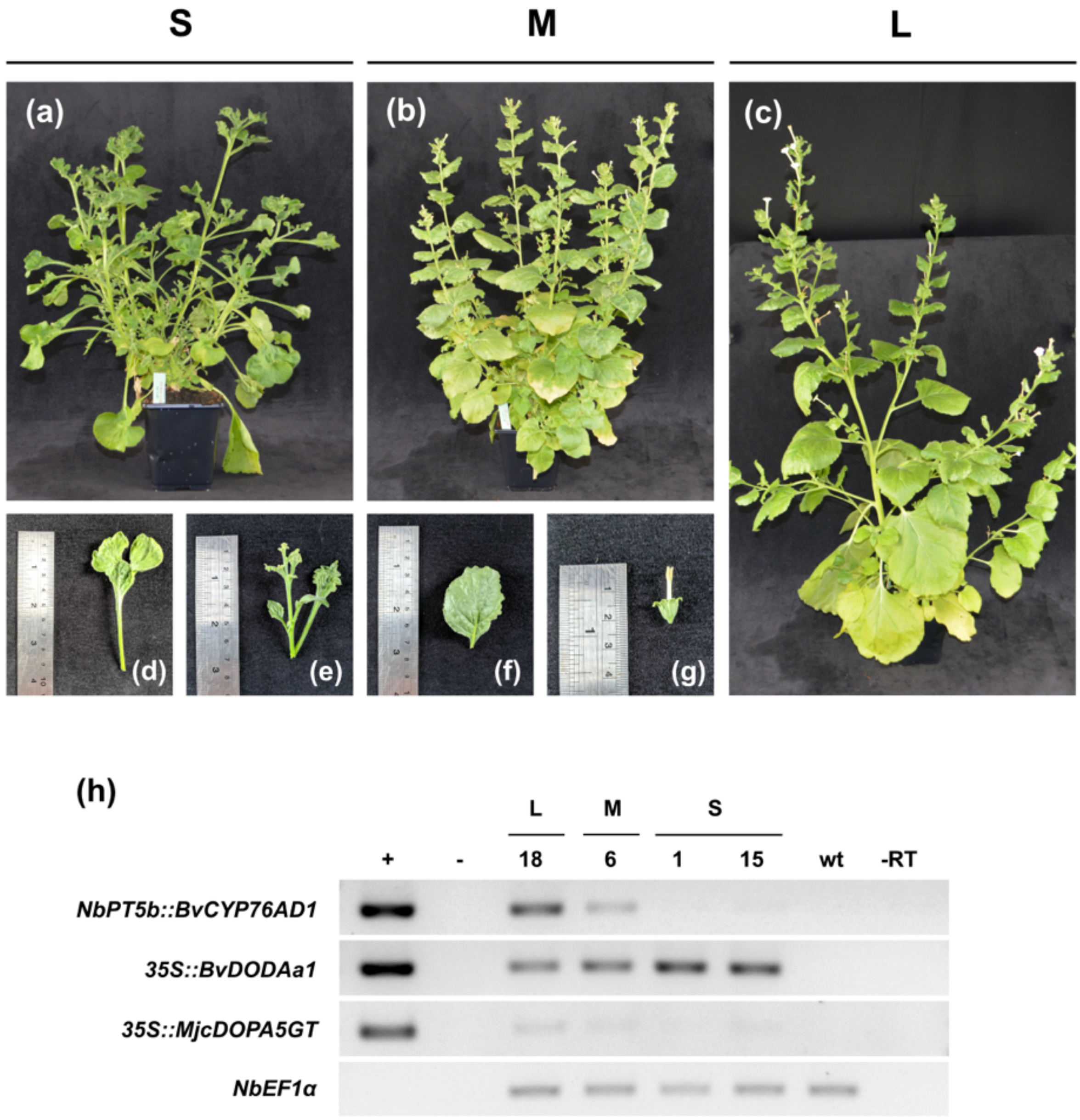

Supplement: S6 Fig — Figure shows examples of T0 plants that varied in the severity of observable developmental defects and were phenotypically classified as severe (S; a, d, and e), moderate (M; b, f, and g), and light (L; c). Only plants with a light phenotype were able to set seeds, although not abundantly. Defects affected overall plant size and leaf and flower morphology. Severely affected plants displayed thicker leaves with strong deformation commonly showing a bifurcated shape (d) and flowers lacking reproductive organs (e). Moderately affected plants displayed altered leaf elongation with curvy edges (f) and flowers with a shorter (or lacking) perianth (g). Light phenotypes included slight leaf deformation, and flowers with shorter perianth but typical overall plant growth (c). (h) Semiquantitative RT-PCR analysis of betalain biosynthetic transgene expression in leaves of N. benthamiana T0 stable NbPT5b-p1 transformants. Line 18 displayed light developmental defects and was able to set seed, line 6 displayed moderate developmental defects, and lines 1 and 15 displayed severe developmental defects. NbEF1α is used as housekeeping control. Positive control (+) is NbPT5b-p1 plasmid DNA. Negative control (−) is water. −RT stands for minus reverse transcriptase control generated from line 18. Original gel images can be found in S4 Data. RT-PCR, reverse transcription PCR. (PDF) [file pbio.3001326.s006.pdf]
