# Supplementary material for: MycoRed: Betalain pigments enable in vivo real-time visualisation of arbuscular mycorrhizal colonisation
Source: PLoS Biol. 2021 Jul 14;19(7):e3001326. doi: 10.1371/journal.pbio.3001326 (PMC8312983; doi:10.1371/journal.pbio.3001326)

**S7 Fig.** One-week old *Nicotiana benthamiana* T1 seedlings from *NbPT5b*-p1 and *NbBCP1b*-p1 expressing lines. *NbPT5b*-p1 lines developed shorter roots and smaller leaves, which also appeared lightly pigmented in some cases. *NbPT5b*-p1 lines 18, 19 and 23, and the *NbBCP1b*-p1 line 32, were able to produce pigments upon colonisation with *R. irregularis*. *NbPT5b*-p1 line 23 didn't produce any pigments upon colonisation.

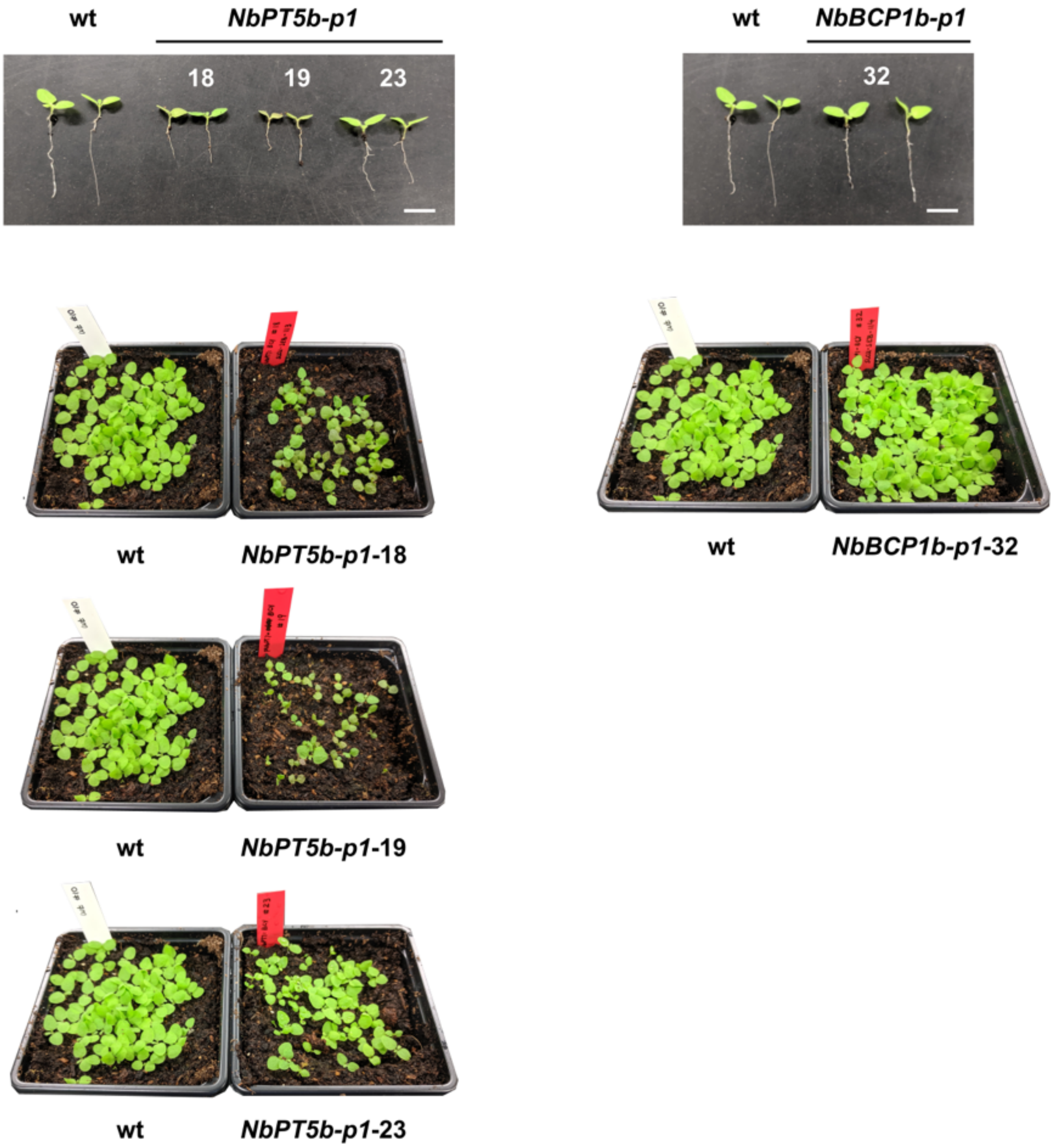

Supplement: S7 Fig — NbPT5b-p1 lines developed shorter roots and smaller leaves, which also appeared lightly pigmented in some cases. NbPT5b-p1 lines 18, 19, and 23, and the NbBCP1b-p1 line 32, were able to produce pigments upon colonisation with R. irregularis. NbPT5b-p1 line 23 did not produce any pigments upon colonisation. (PDF) [file pbio.3001326.s007.pdf]
