# Supplementary material for: MycoRed: Betalain pigments enable in vivo real-time visualisation of arbuscular mycorrhizal colonisation
Source: PLoS Biol. 2021 Jul 14;19(7):e3001326. doi: 10.1371/journal.pbio.3001326 (PMC8312983; doi:10.1371/journal.pbio.3001326)

S8 Fig. Betanin detection by HPLC performed on T1 pigmented leaf tissue of *NbPT5b*-p1 expressing *Nicotiana benthamiana* lines. (a) Some T1 plants show pigmentation in the leaves at varying degrees. (b) Liquid chromatography of pigmented leaves and controls. Pigmented leaves of *NbPT5b*-p1 expressing plants both in inoculated (myc) and non-inoculated (mock) conditions showed a peak at the expected retention time for betanin (red dotted line). Betanin standard is a commercially available *Beta vulgaris* extract and displays two peaks corresponding to the two betanin isomers commonly present in beet extracts. *NbPT5b::GUS* leaf tissue of same age T1 plants was used as negative control. Data underlying this figure can be found in S3 Data.

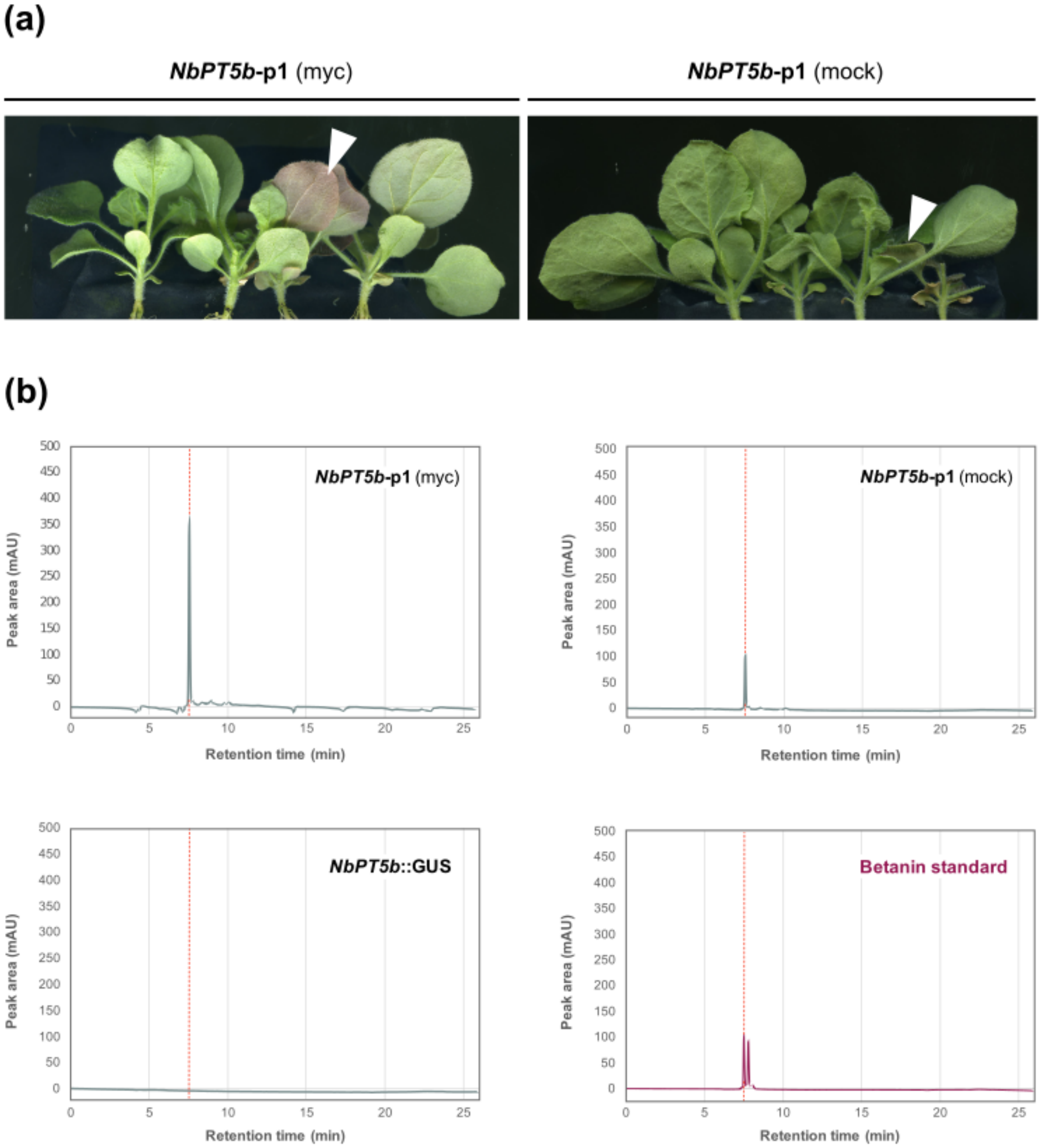

Supplement: S8 Fig — (a) Some T1 plants show pigmentation in the leaves at varying degrees. (b) Liquid chromatography of pigmented leaves and controls. Pigmented leaves of NbPT5b-p1 expressing plants both in inoculated (myc) and non-inoculated (mock) conditions showed a peak at the expected retention time for betanin (red dotted line). Betanin standard is a commercially available Beta vulgaris extract and displays 2 peaks corresponding to the 2 betanin isomers commonly present in beet extracts. NbPT5b::GUS leaf tissue of same age T1 plants was used as negative control. Data underlying this figure can be found in S3 Data. (PDF) [file pbio.3001326.s008.pdf]
