# Supplementary material for: MycoRed: Betalain pigments enable in vivo real-time visualisation of arbuscular mycorrhizal colonisation
Source: PLoS Biol. 2021 Jul 14;19(7):e3001326. doi: 10.1371/journal.pbio.3001326 (PMC8312983; doi:10.1371/journal.pbio.3001326)

S9 Fig. Root systems of *NbPT5b*-p3 (a-d) and *NbBCP1b*-p3 (e-h) *Nicotiana benthamiana* plants colonised by *Rhizophagus irregularis*. Images were taken at 52 dpi. (a,b,e,f) Plants inoculated with *R. irregularis*. (c,d,g,h) Plants descending from the same lines mock inoculated with autoclaved inoculum. (a,c,e,g) are reflective light images, (b,d,f,h) are filtered for red coloring only. Scale bar, 1.3 cm.

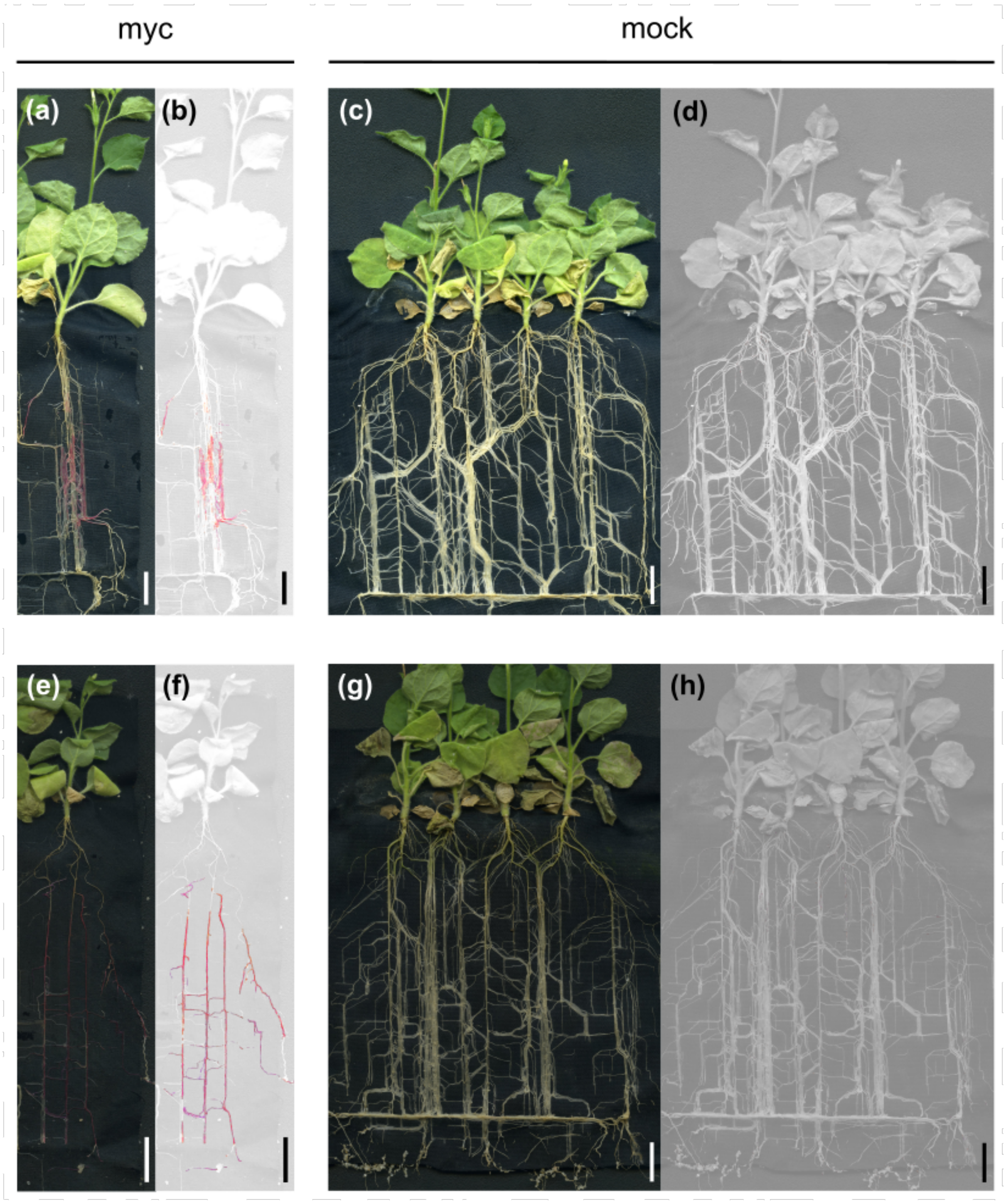

Supplement: S9 Fig — Root systems of NbPT5b-p3 (a–d) and NbBCP1b-p3 (e–h) Nicotiana benthamiana plants colonised by Rhizophagus irregularis. Images were taken at 52 dpi. (a, b, e, and f) Plants inoculated with R. irregularis. (c, d, g, and h) Plants descending from the same lines mock inoculated with autoclaved inoculum. (a, c, e, and g) are reflective light images, and (b, d, f, and h) are filtered for red colouring only. Scale bar, 1.3 cm. dpi, days postinoculation. (PDF) [file pbio.3001326.s009.pdf]
