# Supplementary material for: MycoRed: Betalain pigments enable in vivo real-time visualisation of arbuscular mycorrhizal colonisation
Source: PLoS Biol. 2021 Jul 14;19(7):e3001326. doi: 10.1371/journal.pbio.3001326 (PMC8312983; doi:10.1371/journal.pbio.3001326)

S10 Fig. Quantification of root AM colonisation shows no differences between *Nicotiana benthamiana* WT and reporter lines. *N. benthamiana* plants were inoculated with *Rhizophagus irregularis*, and ink stained 4 weeks after inoculation (wpi). (a) Colonisation extent in *N. benthamiana* WT versus lines expressing *NbBCP1b*-p3 (BCP1-19 and BCP1-24) and *NbPT5b*-p3 (PT5b-16 and PT5b-21). Colonisation extent is calculated as the percentage of root containing arbuscules, vesicles or internal hyphae over the total root system. (b) Frequency of arbuscules and vesicles recorded in colonised roots of *N. benthamiana* WT versus lines expressing *NbBCP1b*-p3 and *NbPT5b*-p3. Frequency is calculated as the percentage of root containing arbuscules or vesicles over the extent of root colonised by *R. irregularis*. Individual points represent individual plants. Error bars represent standard errors. Data underlying this figure can be found in S3 Data.

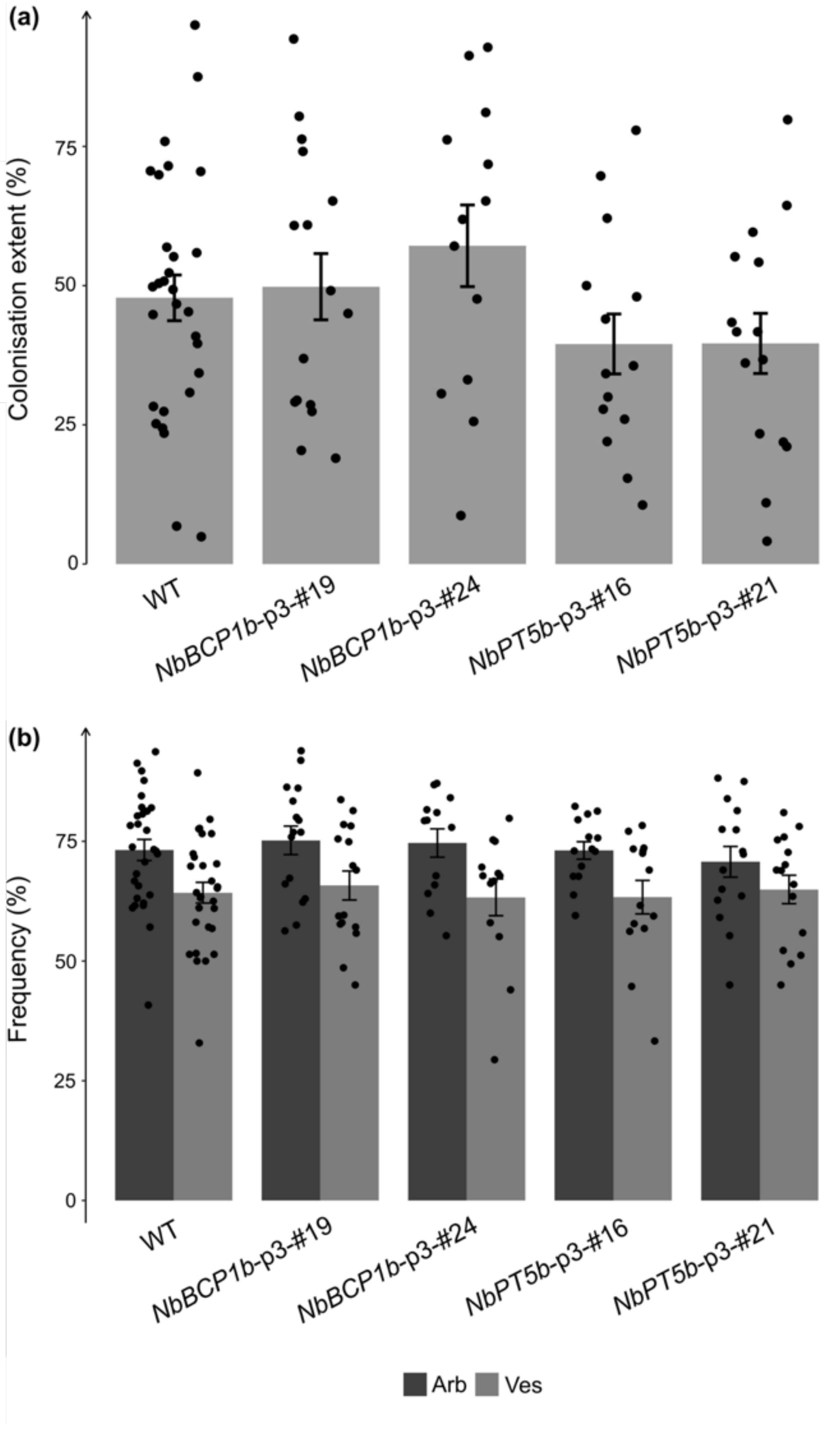

Supplement: S10 Fig — N. benthamiana plants were inoculated with Rhizophagus irregularis and ink stained 4 wpi. (a) Colonisation extent in N. benthamiana WT versus lines expressing NbBCP1b-p3 (BCP1-19 and BCP1-24) and NbPT5b-p3 (PT5b-16 and PT5b-21). Colonisation extent is calculated as the percentage of root containing arbuscules, vesicles, or internal hyphae over the total root system. (b) Frequency of arbuscules and vesicles recorded in colonised roots of N. benthamiana WT versus lines expressing NbBCP1b-p3 and NbPT5b-p3. Frequency is calculated as the percentage of root containing arbuscules or vesicles over the extent of root colonised by R. irregularis. Individual points represent individual plants. Error bars represent standard errors. Data underlying this figure can be found in S3 Data. AM, arbuscular mycorrhiza; wpi, weeks after inoculation; WT, wild-type. (PDF) [file pbio.3001326.s010.pdf]
