# Supplementary material for: MycoRed: Betalain pigments enable in vivo real-time visualisation of arbuscular mycorrhizal colonisation
Source: PLoS Biol. 2021 Jul 14;19(7):e3001326. doi: 10.1371/journal.pbio.3001326 (PMC8312983; doi:10.1371/journal.pbio.3001326)

S11 Fig. The oomycete root pathogen *Phytophthora palmivora* does not induce betalain pigment development in *NbPT5b*-p3 and *NbBCP1b*-p3 *Nicotiana benthamiana* lines within 9 days post infection (dpi). Sixteen transgenic betalain reporter lines per construct were grown on petridishes, eight were not infected (mock) and eight were infected with red fluorescent *P. palmivora* LILI-td zoospores. Plates were subsequently imaged under an epifluorescent microscope at 2 dpi (a) to document pathogen colonisation on roots, and via flatbed scanning at 2 dpi (b) and 9 dpi (c). Application of a red filtering did not show any betalain pigment development. Scale bar, 50 mm.

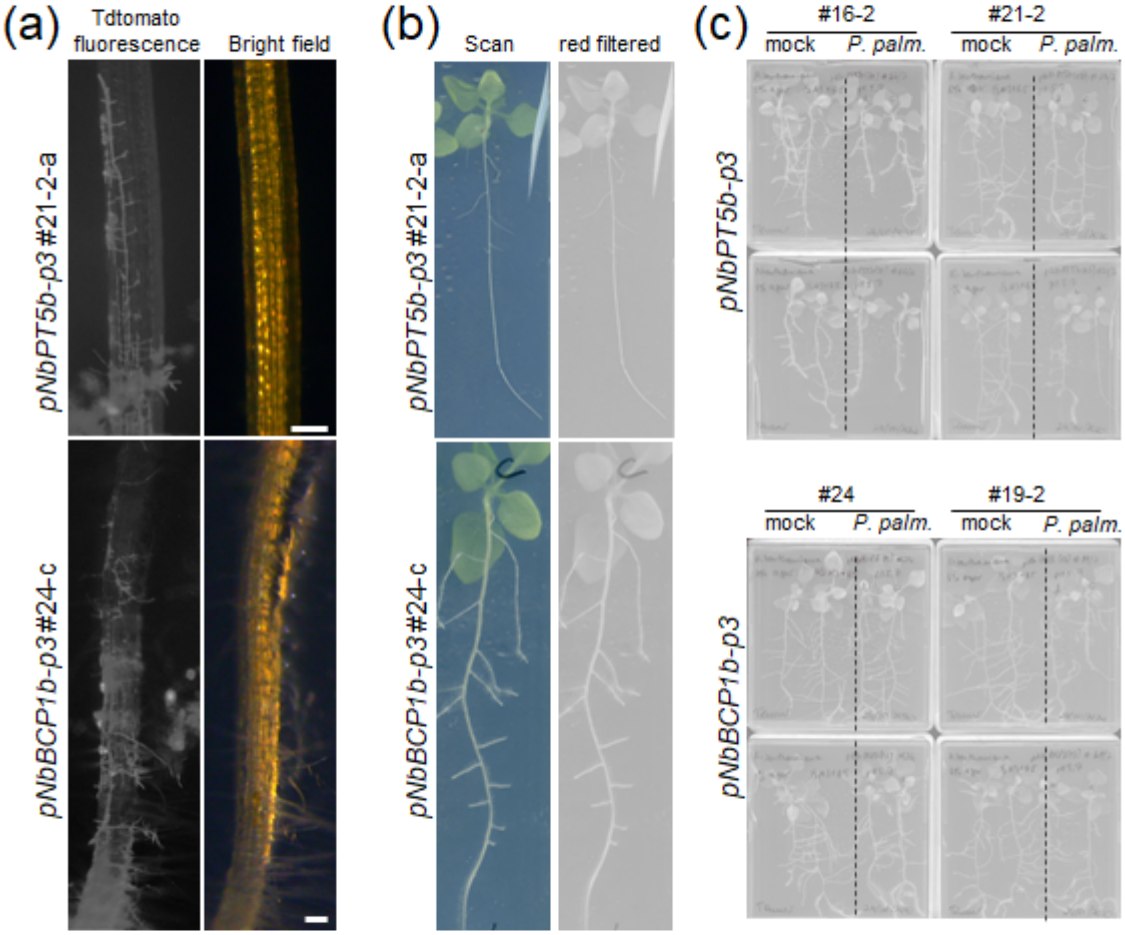

Supplement: S11 Fig — A total of 16 transgenic betalain reporter lines per construct were grown on petridishes: 8 were not infected (mock), and 8 were infected with red fluorescent P. palmivora LILI-td zoospores. Plates were subsequently imaged under an epifluorescent microscope at 2 dpi (a) to document pathogen colonisation on roots and via flatbed scanning at 2 dpi (b) and 9 dpi (c). Application of a red filtering did not show any betalain pigment development. Scale bar, 50 mm. dpi, days postinfection. (PDF) [file pbio.3001326.s011.pdf]
