# Supplementary material for: MycoRed: Betalain pigments enable in vivo real-time visualisation of arbuscular mycorrhizal colonisation
Source: PLoS Biol. 2021 Jul 14;19(7):e3001326. doi: 10.1371/journal.pbio.3001326 (PMC8312983; doi:10.1371/journal.pbio.3001326)

S12 Fig. Betacyanin production in *Medicago truncatula* roots as a response to AM fungi colonisation via expression of the entire betalain pathway under an AM symbiosis specific promoter. (a) Schematic of the multi-gene vector constructed for inducible betalain expression in *M. truncatula* roots with the three betalain biosynthetic genes controlled by the *MtPT4* promoter. (b-e) Expression of *MtPT4*-p3 in roots 4 weeks after inoculation with *Rhizophagus irregularis* led to pigment accumulation in roots. (f-i) Mock inoculated roots expressing *MtPT4*-p3 didn't show any pigment production. (b,f) Images taken under reflective light, (c,g) are filtered for red colouring only. (d,h) Close up root images in bright field, (e,i) and filtered to observe DSRed fluorescence. (j) Root section of *M. truncatula* expressing *MtPT4*-p3. Betalain accumulation correlates with arbuscule formation but can be observed mainly in the endodermal layer adjacent to arbuscule-containing cortical cells, pericycle and steele. Open arrows mark internal hyphae and filled arrows signal cells containing arbuscules.Scale bar (b,f), 1 cm; scale bar (e,i), 1.5 mm; scale bar (j), 150 µm.

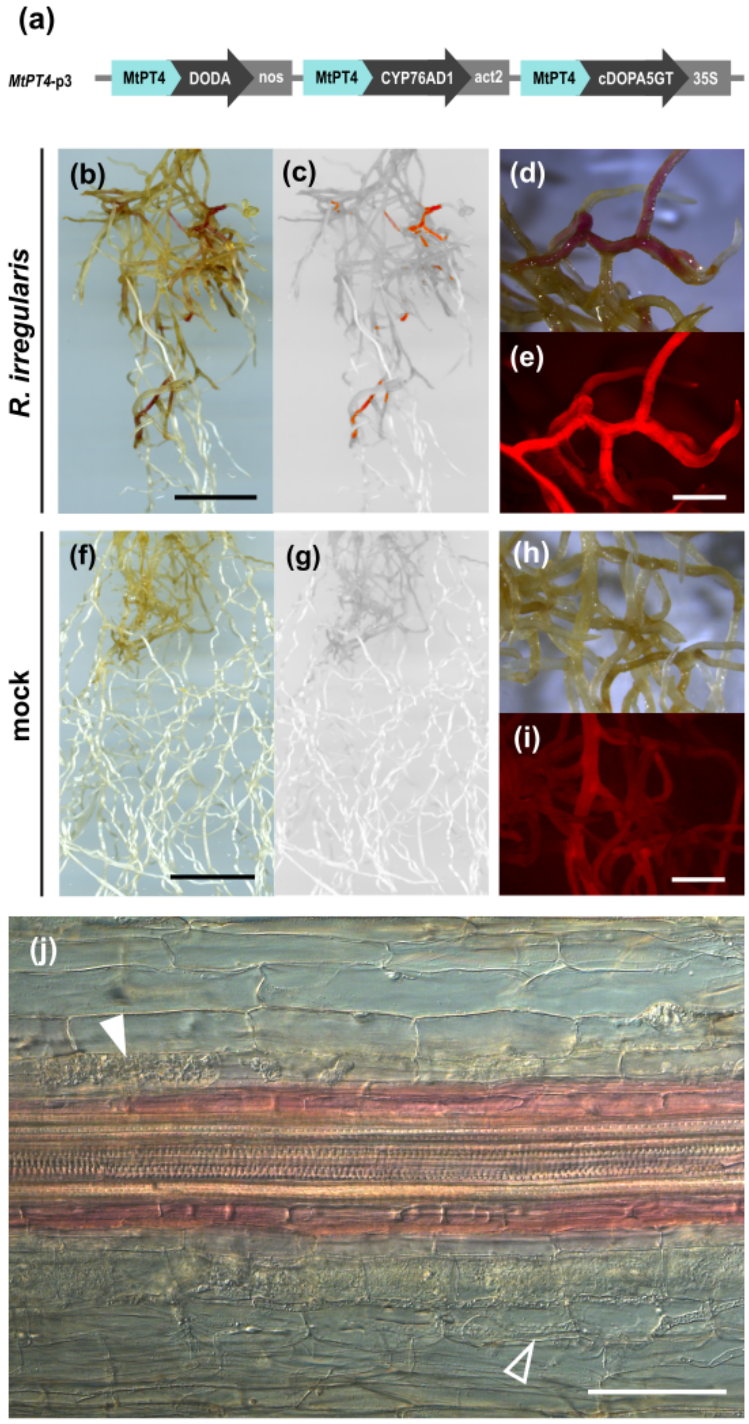

Supplement: S12 Fig — (a) Schematic of the multigene vector constructed for inducible betalain expression in M. truncatula roots with the 3 betalain biosynthetic genes controlled by the MtPT4 promoter. (b–e) Expression of MtPT4-p3 in roots 4 wpi with Rhizophagus irregularis led to pigment accumulation in roots. (f–i) Mock inoculated roots expressing MtPT4-p3 did not show any pigment production. (b and f) Images taken under reflective light, and (c and g) are filtered for red colouring only. (d and h) Close up root images in bright field, and (e and i) and filtered to observe DSRed fluorescence. (j) Root section of M. truncatula expressing MtPT4-p3. Betalain accumulation correlates with arbuscule formation but can be observed mainly in the endodermal layer adjacent to arbuscule-containing cortical cells, pericycle, and steele. Open arrows mark internal hyphae and filled arrows signal cells containing arbuscules. Scale bar (b and f), 1 cm; scale bar (e and i), 1.5 mm; scale bar (j), 150 μm. AM, arbuscular mycorrhiza; wpi, weeks after inoculation. (PDF) [file pbio.3001326.s012.pdf]
