# Supplementary material for: MycoRed: Betalain pigments enable in vivo real-time visualisation of arbuscular mycorrhizal colonisation
Source: PLoS Biol. 2021 Jul 14;19(7):e3001326. doi: 10.1371/journal.pbio.3001326 (PMC8312983; doi:10.1371/journal.pbio.3001326)

S13 Fig. Hairy roots of *dmi3* mutant *Medicago truncatula* plants expressing *MtPT4*-p3 do not produce any perceptible betalain colouration 4-weeks after inoculation with *Rhizophagus irregularis*. (a-b) Example of an A17 *M. truncatula* root system expressing *MtPT4*-p3 able to produce betalains upon inoculation. (c-d) Example of *dmi3 M. truncatula* root system expressing *MtPT4*-p3. (a,c) Images taken under reflective light, (b,d) are filtered for red colouring only. Scale bar, 1 cm.

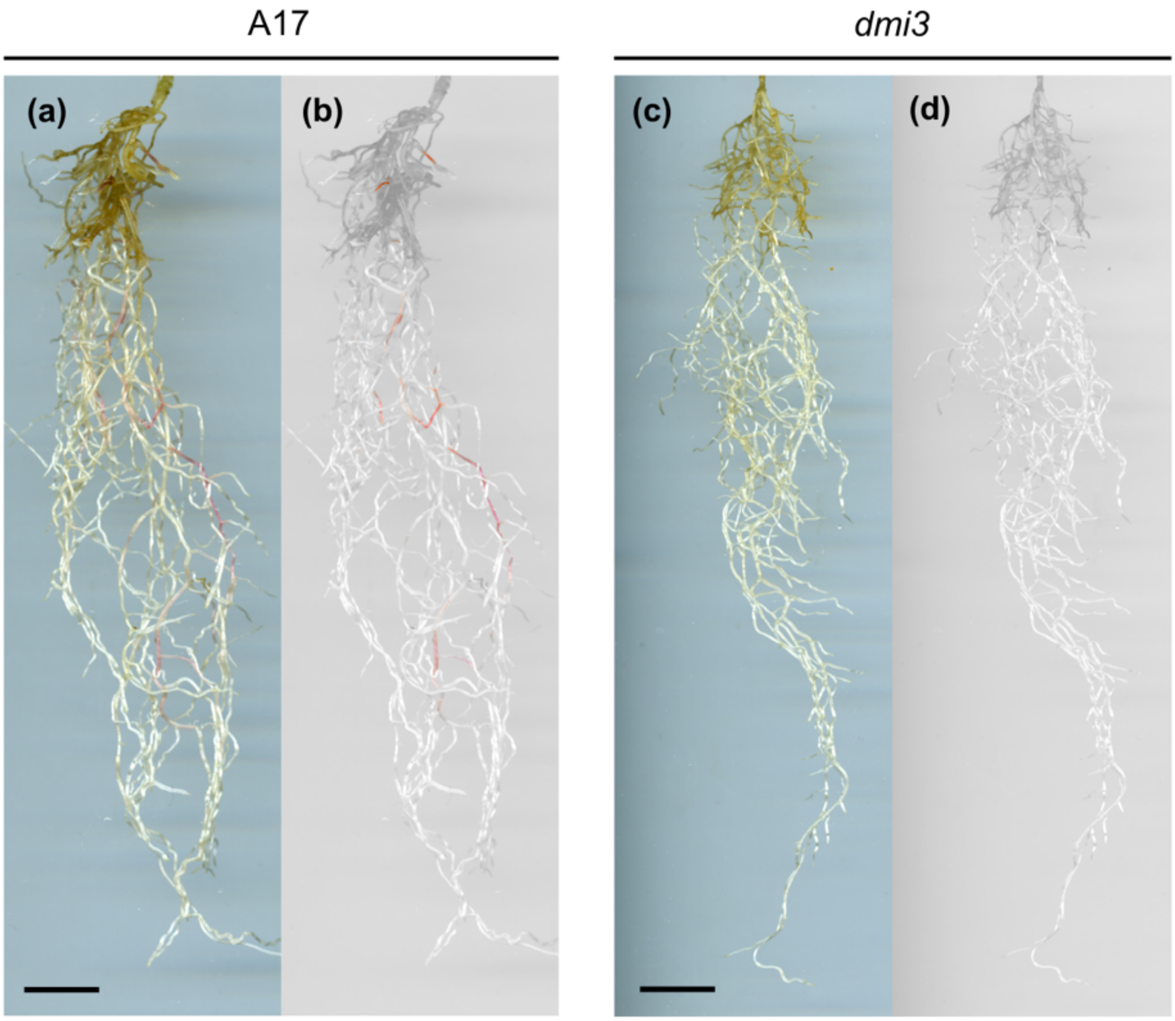

Supplement: S13 Fig — (a and b) Example of an A17 M. truncatula root system expressing MtPT4-p3 able to produce betalains upon inoculation. (c and d) Example of dmi3 M. truncatula root system expressing MtPT4-p3. (a and c) Images taken under reflective light, and (b and d) are filtered for red colouring only. Scale bar, 1 cm. wpi, weeks after inoculation. (PDF) [file pbio.3001326.s013.pdf]
