# Supplementary material for: MycoRed: Betalain pigments enable in vivo real-time visualisation of arbuscular mycorrhizal colonisation
Source: PLoS Biol. 2021 Jul 14;19(7):e3001326. doi: 10.1371/journal.pbio.3001326 (PMC8312983; doi:10.1371/journal.pbio.3001326)

S14 Fig. (a) Schematic of the fungal visualisation and quantification process of *Medicago truncatula* hairy roots after inoculation with *Rhizophagus irregularis*: 1, *M. truncatula* transgenic hairy roots express the DSRed marker and can be selected through detection of fluorescence under 510-560 nm; 2, DSRed positive roots are cut and divided into betalain producing and non-producing root fragments; 3, Pigmented and non-pigmented root fragments are ink stained separately (betalain colouration fades upon incubation with potassium hydroxide during the ink staining process); 4, Ink stained root fragments are mounted in glass slides and imaged for visualisation and quantification of fungal structures. (b) Example of *MtPT4*-p3 pigmented root fragments showing fungal colonisation. (c) Example of *MtPT4*-p3 non-pigmented uncolonised root fragments. Scale bar, 100 μm.

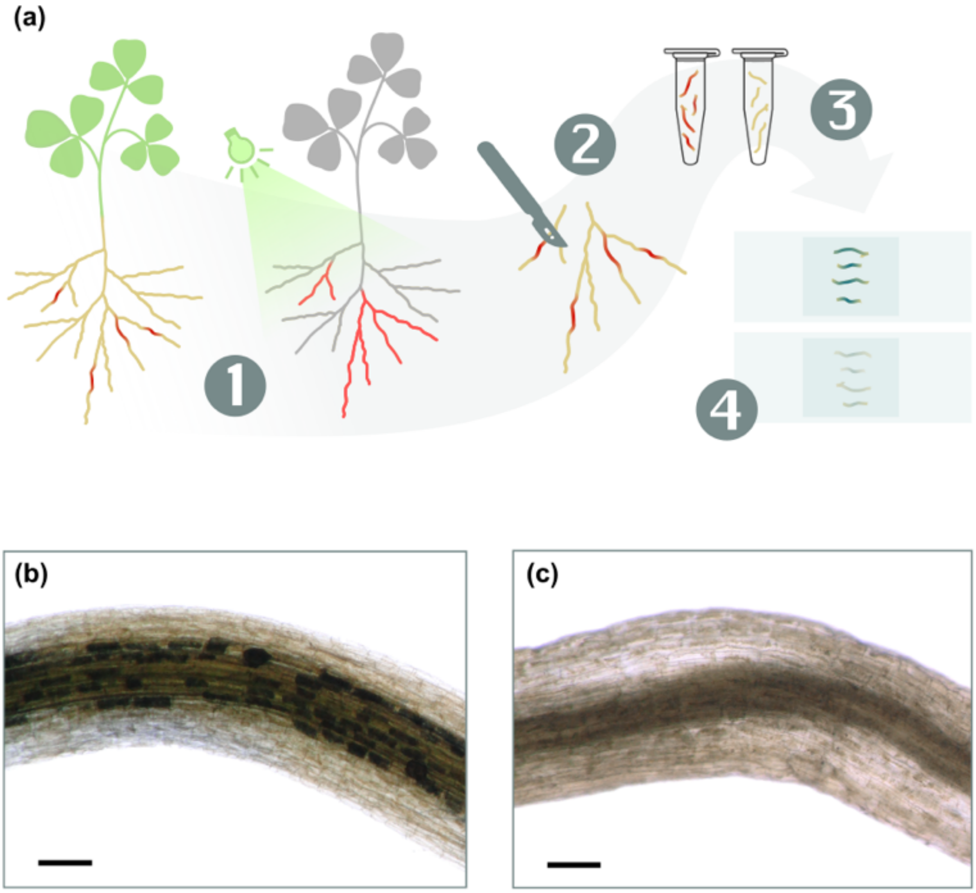

Supplement: S14 Fig — (a) Schematic of the fungal visualisation and quantification process of Medicago truncatula hairy roots after inoculation with Rhizophagus irregularis: (1) M. truncatula transgenic hairy roots express the DSRed marker and can be selected through detection of fluorescence under 510–560 nm; (2) DSRed positive roots are cut and divided into betalain producing and nonproducing root fragments; (3) pigmented and nonpigmented root fragments are ink stained separately (betalain colouration fades upon incubation with potassium hydroxide during the ink staining process); and (4) ink-stained root fragments are mounted in glass slides and imaged for visualisation and quantification of fungal structures. (b) Example of MtPT4-p3 pigmented root fragments showing fungal colonisation. (c) Example of MtPT4-p3 nonpigmented uncolonised root fragments. Scale bar, 100 μm. (PDF) [file pbio.3001326.s014.pdf]
