# Supplementary material for: MycoRed: Betalain pigments enable in vivo real-time visualisation of arbuscular mycorrhizal colonisation
Source: PLoS Biol. 2021 Jul 14;19(7):e3001326. doi: 10.1371/journal.pbio.3001326 (PMC8312983; doi:10.1371/journal.pbio.3001326)

S15 Fig. *NbPT5b* promoter-controlled betalain biosynthesis allows for dynamic tracing of root colonisation processes in *Nicotiana benthamiana*. Transgenic *NbPT5b*-p3 plants grown in a rhizotron setup supplied with *Rhizophagus irregularis* spore inoculum imaged over time. (a) Reflective light images, (b) represent the same images filtered for red/magenta hues. dpi, days post inoculation. Scale bar, 1 cm.

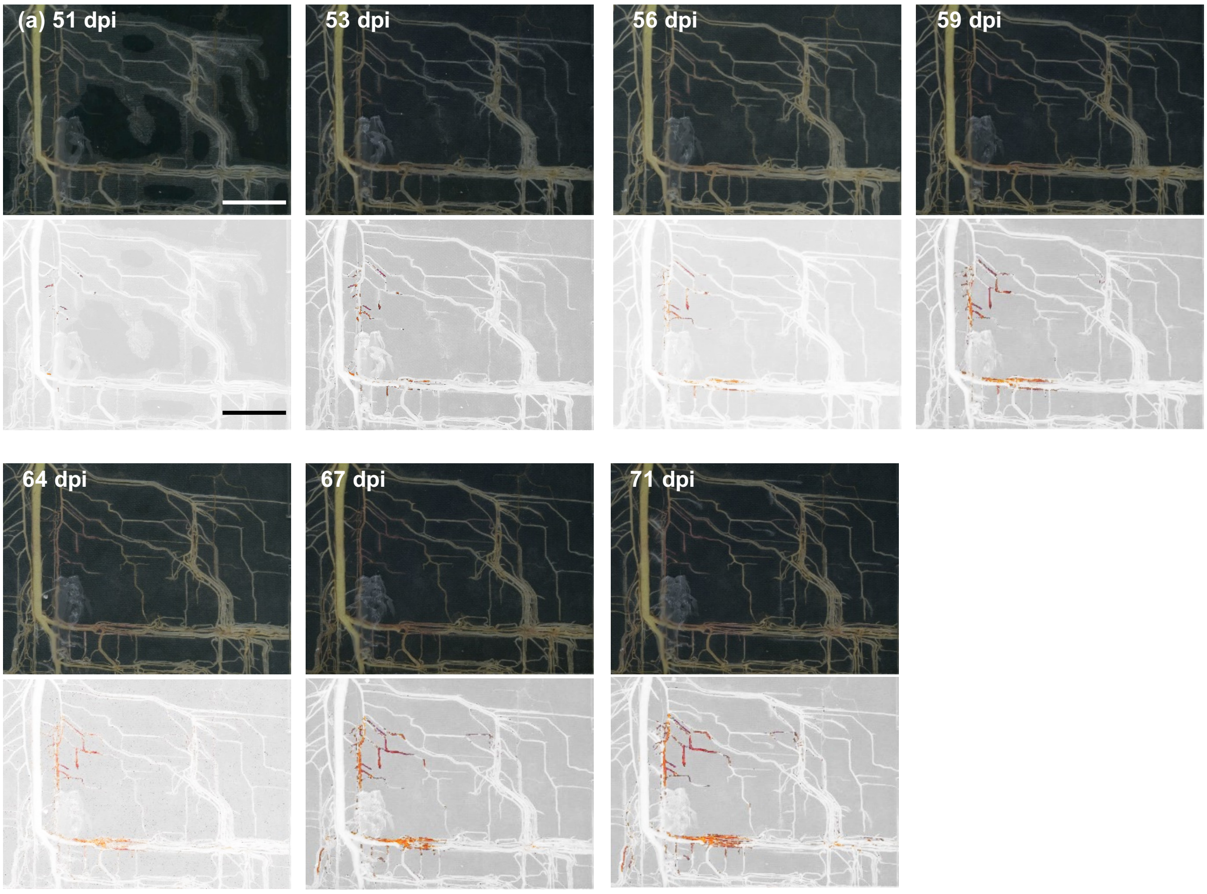

Supplement: S15 Fig — Transgenic NbPT5b-p3 plants grown in a rhizotron setup supplied with Rhizophagus irregularis spore inoculum imaged over time. (a) Reflective light images, and (b) represent the same images filtered for red/magenta hues. Scale bar, 1 cm. dpi, days postinoculation. (PDF) [file pbio.3001326.s015.pdf]
