# Supplementary material for: MycoRed: Betalain pigments enable in vivo real-time visualisation of arbuscular mycorrhizal colonisation
Source: PLoS Biol. 2021 Jul 14;19(7):e3001326. doi: 10.1371/journal.pbio.3001326 (PMC8312983; doi:10.1371/journal.pbio.3001326)
Supplement: S1 Table — We selected roots for DSRed fluorescence and then cut and divided them in pigmented (red rows) and nonpigmented (noncoloured rows) fragments for ink staining. N refers to the total number of root fragments analysed for each condition. Table shows the number of root fragments containing fungal structures and the average extent of colonisation by these structures over the length of the root fragment. Error shown as standard error. %AC, percentage of arbuscule colonisation; %IHC, percentage of internal hyphae colonisation; %VC, percentage of vesicle colonsation; A, arbuscules; IH, internal hyphae; V, vesicles; wpi, weeks postinoculation. (PDF) [file pbio.3001326.s016.pdf]

S1 Table. Quantification of fungal structures observed in hairy roots of *Medicago truncatula* expressing *MtPT4*-p3 4 weeks post-inoculation with *Rhizophagus irregularis*. We selected roots for DSRed fluorescence, and then cut and divided them in pigmented (red rows) and non-pigmented (non-coloured rows) fragments for ink staining. N refers to the total number of root fragments analysed for each condition. Table shows the number of root fragments containing fungal structures, and the average extent of 18olonization by these structures over the length of the root fragment. IH, internal hyphae; A, arbuscules; V, vesicles; %IHC, percentage of internal hyphae 18olonization; %AC, percentage of arbuscule 18olonization; %VC, percentage of vesicle 18olonization. Error shown as standard error.

Genotype	Inoculum	Pigmented	N	Percent of colonized root fragments (%)			Average length of root fragments colonised (%)		
				IH	A	V	%IHC	%AC	%VC
A17	+	+	25	96.0	96.0	84.0	65.0 ± 6.4	63.3 ± 6.3	35.9 ± 5.4
		-	45	35.6	35.6	13.3	8.8 ± 2.6	8.8 ± 2.6	2.4 ± 1.1
	-	-	10	0.0	0.0	0.0	0.0	0.0	0.0
dmi3	+	+	0	0.0	0.0	0.0	0.0	0.0	0.0
		-	10	0.0	0.0	0.0	0.0	0.0	0.0
	-	-	10	0.0	0.0	0.0	0.0	0.0	0.0
