# Supplementary material for: MycoRed: Betalain pigments enable in vivo real-time visualisation of arbuscular mycorrhizal colonisation
Source: PLoS Biol. 2021 Jul 14;19(7):e3001326. doi: 10.1371/journal.pbio.3001326 (PMC8312983; doi:10.1371/journal.pbio.3001326)
Supplement: S2 Table — (PDF) [file pbio.3001326.s017.pdf]

S2 Table. List of oligonucleotide primers used in this study.

Primer ID	Sequence 5'-3'	Purpose
MtPT4pro_F	CTCGATCCACAACAAAGATT	Cloning of MtPT4 promoter from gDNA
MtPT4pro_R	CTCAAGTTGGTTTTTGGAGT	
MtBCP1pro_F	CACATCTAGAGAGAGGGAGATGTGTT	Cloning of MtBCP1 promoter from gDNA
MtBCP1pro_R	TCTCGGATCCTGCAATTGCAACTGATGAAAG	
NbPT5bpro_F	GTGAGAAATTTACCAAATACCTATAAATAATGTATCAGC	Cloning of NbPT5b promoter from gDNA
NbPT5bpro_R	GTTTGCTTTTTACTGCAAATTTTGGTTG	
NbBCP1bpro_F	ACTGGTATTAGAATAATAGCCTGTTTAGCC	Cloning of NbBCP1b promoter from gDNA
NbBCP1bpro_R	GGAAAATTCTTCTTTAAGGGTTAGTTG	
NbPT5b_qF	TTACTACGATCCTGCTACACACG	qRT-PCR for gene validation
NbPT5b_qR	CAGTTAAAGTACCAACCAAAGCC	
NbBCP1b_qF	TCGAAGATGGAGTTCCGGCA	qRT-PCR for gene validation
NbBCP1b_qR	AATCAACATCAATGGTCCAGCC	
RiBTub_qF	AACAATTGGGCCAAAGGTCACT	qRT-PCR for gene validation
RiBTub_qR	CGCTTCTTTGCGAACAACATCT	
RiEF_qF	TGTTGCTTTCGTCCCAATATC	qRT-PCR for gene validation
RiEF_qR	GGTTTATCGGTAGGTCGAG	
NbEF1a_qF	AGCTTTACCTCCCAAGTCATC	qRT-PCR for gene validation and RT-PCR of T1 *N. benthamiana* plants
NbE1Fa_qR	AGAACGCCTGTCAATCTTGG	
MtSTR_qF	TTCCAATGATGCAGTCCCA	qRT-PCR for gene validation
MtSTR_qR	TGGTTATGACTGCAAATGTGAG	
MtRAM2_qF	GGGGGCTGATATGGTTTTGGGA	qRT-PCR for gene validation
MtRAM2_qR	TGTAACCCTCCTTGCACAAAGC	
MtUBQ_qF	GCAGATAGACACGCTGGGA	qRT-PCR for gene validation
MtUBQ_qR	AACTCTTGGGCAGGCAATAA	
BvCYP76AD1_qF	AACTGCAACAACAACGACGA	RT-PCR of T1 *N. benthamiana* plants
BvCYP76AD1_qR	AAAATGTCGACGAGCAAATGG	
BvDODAa1_qF	TGCTGCTATGTACCAGTTCAAGT	RT-PCR of T1 *N. benthamiana* plants
BvDODAa1_qR	CAGTTTCCGCCGTTTCGAAA	
MjcDOPA5GT_qF	CAACATTCATCATCTGATAA	RT-PCR of T1 *N. benthamiana* plants
MjcDOPA5GT_qR	GTGATCCAAATGAGATGTAT	
MtPT4_lv0_F	TGAAGACATGGAGCTCGATCCACAACAAAGATT	MoClo Golden gate cloning of promoters
MtPT4_lv0_R	TGAAGACATCATTCTCAAGTTGGTTTTTGGAGT	
MtBCP1_lv0_F	TGAAGACATGGAGAATGCGTTGCTTAAGAGTTG	MoClo Golden gate cloning of promoters
MtBCP1_lv0_R	TGAAGACATCATTTGATCAAAATTTGATTTTGG	

Primer ID	Sequence 5'-3'	Purpose
NbPT5b_lv0_F	TGAAGACATGGAGGTGAGAAATTTACCAAATACCTATA AATAATGTATCAGC	MoClo Golden gate cloning of promoters
NbPT5b_lv0_R	TGAAGACATCATTGTTTGCTTTTTACTGCAAATTTTGGT TG	
NbBCP1b_lv0_F	TGAAGACATGGAGACTGGTATTAGAATAATAGCCTGTT TAGCC	MoClo Golden gate cloning of promoters
NbBCP1b_lv0_R	TGAAGACATCATTGGAAAATTCTTCTTTAAGGGTTAGTT G	
