# Supplementary material for: MycoRed: Betalain pigments enable in vivo real-time visualisation of arbuscular mycorrhizal colonisation
Source: PLoS Biol. 2021 Jul 14;19(7):e3001326. doi: 10.1371/journal.pbio.3001326 (PMC8312983; doi:10.1371/journal.pbio.3001326)
Supplement: S3 Table — All vectors are available to obtain free of charge through Addgene. Plasmid maps and sequence files can be downloaded in the Addgene website with the catalogue numbers provided. (PDF) [file pbio.3001326.s018.pdf]

S3 Table. List of vectors used in this study. Level 0 vectors used for construction of level 2 multigene vectors, which are also listed as MycoRed reporter constructs. All vectors are available to obtain free of charge through Addgene. Plasmid maps and sequence files can be downloaded in the Addgene website with the catalog numbers provided.

Level 0 vectors

Plasmid ID	Addgene #	Description
pL0-MtPT4Pro	159414	*Medicago truncatula* PT4 promoter sequence (836 bp immediately upstream of start codon) in pICH41295 MoClo Golden Gate level 0 acceptor for Pro+5U modules.
pL0-MtBCP1Pro	159415	*Medicago truncatula* BCP1 promoter sequence (1108 bp immediately upstream of start codon) in pICH41295 MoClo Golden Gate level 0 acceptor for Pro+5U modules.
pL0-NbPT5bPro	159416	*Nicotiana benthamiana* PT5b promoter sequence (1068 bp immediately upstream of start codon) in pICH41295 MoClo Golden Gate level 0 acceptor for Pro+5U modules.
pL0-NbBCP1bPro	159417	Nicotiana benthamiana BCP1b promoter sequence (1231 bp immediately upstream of start codon) in pICH41295 MoClo Golden Gate level 0 acceptor for Pro+5U modules.
pL0-BvDODAα1	162528	*Beta vulgaris* 4,5-DOPA dioxygenase alpha 1 coding sequence in pICH41308 MoClo Golden Gate level 0 acceptor for CDS1 modules.
pL0-BvCYP76AD1	162529	*Beta vulgaris* cytochrome P450 76AD1 coding sequence in pICH41308 MoClo Golden Gate level 0 acceptor for CDS1 modules.
pL0-MjcDOPA5GT	162530	*Mirabilis jalapa* cyclo-DOPA 5-O-glucosyltransferase coding sequence in pICH41308 MoClo Golden Gate level 0 acceptor for CDS1 modules.

MycoRed reporter vectors

Plasmid ID	Addgene #	Description
NbPT5b-p3	160001	Multigene construct containing the betalain pigment biosynthetic genes under the control of the NbPT5b promoter for visualisation of arbuscular mycorrhizal colonisation in *Nicotiana benthamiana* roots.
NbBCP1b-p3	160002	Multigene construct containing the betalain pigment biosynthetic genes under the control of the NbBCP1b promoter for visualisation of arbuscular mycorrhizal colonisation in *Nicotiana benthamiana* roots
MtPT4-p3	160003	Multigene construct containing the betalain pigment biosynthetic genes under the control of the MtPT4 promoter for visualisation of arbuscular mycorrhizal colonisation in *Medicago truncatula* roots.
