# Supplementary material for: MycoRed: Betalain pigments enable in vivo real-time visualisation of arbuscular mycorrhizal colonisation
Source: PLoS Biol. 2021 Jul 14;19(7):e3001326. doi: 10.1371/journal.pbio.3001326 (PMC8312983; doi:10.1371/journal.pbio.3001326)
Supplement: S1 Data — All sequences were selected immediately upstream the start codon of the gene. (PDF) [file pbio.3001326.s019.pdf]

S1 Data. AM specific promoter sequences used for vector construction. All sequences were selected immediately upstream the start codon of the gene.

>MtPT4 promoter (836 bp)

CTCGATCCACAACAAAGATTAATTTTTGTTCAAACAACTAATACTATCTGAAAATACACTTCCAAAAATATGTTAAATTTTTG
TTTTTTTTAACATGGAGTGAAAAAAAGTTGCTCCCTTATTATTTAATTGGCACACATTTTTGTATGTTTAGTAAATGACACGC
ATTCTATTCGGTGGTAACGTTTGTCCTCACATACACCTCATTTGAATTGCATTTGTATCCTACTACCTCCCTTTCCAATTATA
TAAGATATTTTTGAATTTTTTTTGTTCCTTTTTATAAGACCTCCTTGTTATTTCCAATTAAATTAATTATTTCTTTACATATA
TGTCCCTATTTATTATACATTTTTTTCTTCAATCAGCAATAAATAGCATGTTGAAAATATAAACTATCTCTCTTTCTAAAGGA
TAAAATTGTAAAATCAATAGTGATTACAAATAACTTTAATACAAATACTCACTTTCTTAATTCCTGTGATTTTGGCAAAAAGG
TCTTGTATATAGGGACGGAGGTAGTATTATGTTAGCAAGCAGCTAAAAAACAAAACAATAATCCTCTTTTAACAGCTAACTGT
AACCAAAACAAAAGCACTTAGCATTTTTGAACATTCCCTATAAGAATATCCTAATTAATTGATCAACACTAGGTGAATATCCA
TAACATTTAGCTAGCAATCTCAATTTTTCTTGTTCTCATCTCCTAAGCTAACTTAGGACTAAACGTCACATTGTTACATTATC
AAACACATATCAATGCATAGCCATAATAATATATATAAGAGCATACACATAAACACCTAAACCAAATTAACTCCAAAAACCAA
CTTGAG

>MtBCP1 promoter (1108 bp)

AATGCGTTGCTTAAGAGTTGGTTAATCTATTTGAAATGTTGCGATTATAGTAGATCTTGTGCTCCCATCTTTAATATATCTTT
TACACTTACTCCTTAGTTATACAAACATTAAACATCATAACTTCTAAGGTAATTGATACGTGCCAAATTTTCTAAACTCCATT
AAATTTGGTTTGAAAGAAATTTCATGAAATTCCTCTAGTTCGTTATAAATATTAGATAATTAGGTTTTGAGTAGCATTGTAGC
TACCATGACCTATTTCCATTACATTTGTAGGTTTAAAAGATCAAAGATGACTTGGGGTATCAATTAACGAGTGAGCATATATG
CGAGCAAGGAAAGAGATAAGTCAGAGTGTTTAAGGAAAGCAAGGCAAGAAAATTCACTTAGGGAGCCAAAACAAGGTCAATAC
TCGATTACATCTCTAATTTAAGCTTGAGAACTTGATATATTCGAGACTCCTCAAGTATTTGTACTCCAAGTGTCAATACTCGA
TTACATCTCTAATTTTCCAAATAAAGAAACTCGTAAGAAACAAACATTATTTATGTGTAAACTTTCTGCATGGAGACAACTCA
ATTATAACGAAGCCCGGAAGAGGAACTATATATACTGTTTCTATTTCCCTTTGACACGGTCCACACTATATTGCATACTTGTT
TACTGCCAAATAAAAACAAGTATGTCCACGTGGTTAAATAAAAACAAAAGAAAACTTTCTTTTTGTTCAAGTGATAGAAAGTT
TCTTTACATTAATATAAAATAATATAAGTGTATTCCAAAAAGGTAAAAAAAAAAATAATAAGTTGTGGAAATTCAACCTCGTC
TTAGTAAAAAGCCGACCAAATCCGGTAGCCGTTTATAATAGCAAACAAATATTCTATTATTGTTGTGACGATAAAATCAATAC
ACCCACACATGACAACTATATATAGCCTAAGTTGATATGCACAATTCATCAAATCAAATCAAAACAATTACATTTGCTAACTT
GCTATTCCTTTGATTGAGATTTTATTCTAGGAAATAAAATTTTGACACTCATTTTTTTAAAGTTAATTTCTACATTTAGAGAG
AAATATTTTCCAAAATCAAATTTTGATCA

>NbPT5b promoter (1068 bp)

GTGAGAAATTTACCAAATACCTATAAATAATGTATCAGCCGTATATAAATAACGTATAAGAGATGTTTATACACTATTATATA
TTACTTATATAATGTTATACATTATTATACACTATTATACAAATGAATCATATCAATGGAGCTTCACTGATTCTTCTTCTTCT
TTGGGCATCTTCTGAAATTCAACTCAAATCTACCTCAAATCTGCTCCAAATTATTTTAAATTTGAGTTTTGAACTTCTCTTGA
CGATCCCAATCAATTGGGCCAACACCTAATCCAAACAACTAACAAAATCGAAAAATCTAATTTTAAAATTGAAGCTTCAAATG
ACCTTTAATGGTGACTCCAAAAATAATAATTCTCTTACGTGGTATTTTTTCTTCTCATCTTAATAGTGATTATCAATTTTGAA
TATGGAAGGAGAATTAAAAAATATATATAATGGTAGGGAAACTTTGGGGTGCAAAGTCGTGAAAGAAGAGGAAGAAGAAGAAC
GAAGAAAAGAAAAAAAATAGGGTGGGCTAACTGGTGAAATTTTTTTCCAAATTATATATAACCTAGGCTATATCATGTAAGAAC
TTCAAATGTGGGCCATTTTTTGATGGATTGTTGGACCAAATGACAAGGAGCGTAATTCCCCCTTCATTTTATATGGCACACTA
GGATATTATGAGTTTAATAAATAAAGAATTTTTTTTATGATTTTAAACATGTCGTGACTATAAAATTGTGTTATTAAAGTAAA
CTAAGAAGTTTGAAGTTAGATTATATCAATTATATAGATTTGTTTTTTTTTTGACACCAACTAACAAGAAAAAGTTGTCATAT
AAAGCATAAGCATGCGCGCTTTTTGTCCTAGGTTATTTTTGATTGAAGCATATGCTTGATTTAAGCAAAGAAGAAGGATATCC
TGTGGATACTTATCCTCAATTTCTTGTTGTAGCCACTGAGCGCTTAGGATTAAACGTCATTTAATTCACATTGTGCGCTGAGT
CTATATATGGGAGTTACATACACCAAAACAGTACCATGAAAACACAACCAAAATTTGCAGTAAAAAGCAAAC

>NbBCP1b promoter (1231 bp)

ACTGGTATTAGAATAATAGCCTGTTTAGCCAAAAATTGAAGTGTTTGGTTTTGGAAGGAAAAAAAAGTATTTTGAGGAGAGGC
AGAAGCAGAAAAAAGTAGCTTCTCTCCAAAAGTACTTTTTTGAGAAGCATTTTTGAGAAAAATACACTTAGAAGCAGTTTTTT
AAAGCTTGGCCAATCACTAATTGCTGCTCAGAAGTGCTTTTCAAACTAATTAGCCAAACACAAACTACTTCTCATCAAAAGTA
CTTTCGAAAAAAACACTTATTAAAGTAAGCTAATTTTTGCAGCTTGGCTAAATGGATTATAAATCACGTATCTGTAAAACTAC
AAATTATATTTTATTGTTCCTCACATTTTTCGAGGTAAACATAAATATAAGTAAAATATAATGGTAATGTACTCCACTTGCTG
AATAACTTAGTGTTTCATAGCAAGCATATGCTCCACAAATTAAGAGTATGGAGCCATAATATTTCCAATATGAGTGACCCTAT
AGTTACAATAACAACAACAATCCAGTATAATCTCACATAGTGGGGTCTGGTGAGGGTAGTATGTACGTAGACCTTATCTCTAT
CCTGGGGTAGAGAGGCTGTTTCCAAATAGACTCCCGACATCATTCTCTCCAAGAACTTCTCACCTTGCTCTTGGGGAAACTCG
AACTCACAACCTCTATAGTTAAAACATGATAAATTATGGTCATATTTGTATTCTCTTTTTTTGCTTTAATTTTGGGTATGATA
AAATTCCTTATAAATGCTAACAACCTACTCCGTCCTGTTCCAAATAGTAATCGTTTTAGCATGCTGAAATTGTTTCAAAATAT
TTGGCATTTTAGAAAAATAAAATATATTTGTTATTTTTTTATTAACCTTACTACTCCAATTAGTCGATTGTTAATAAGATGAG
ACATTTAAGAGAAAAAAACAACGATAAGTTAGACAAATAATCTTATAATTAAATATGTCAAAAATCATAAAAGACAAATAATA
TGGAATAGAGTAAATATCTACCTACAATACTAAGGTTTTCTTCTCCTTTGAACTTTACGAGAATAATTAGTGGAATATTTGCC
AGCCTAACTCATTGGAATTCACGTACTAGAATTCAAATTAATGAATCACACTTCGTTATCCTCGGATCTCGCTAAGCTCTCTT
GTTAAGTTAAGGTATAAATAGCCATACCAATTTTTCTTTTTTCAACTAACCCTTAAAGAAGAATTTTCC
