# Supplementary figures and images for: MycoRed: Betalain pigments enable in vivo real-time visualisation of arbuscular mycorrhizal colonisation
Source: PLoS Biol. 2021 Jul 14;19(7):e3001326. doi: 10.1371/journal.pbio.3001326 (PMC8312983; doi:10.1371/journal.pbio.3001326)

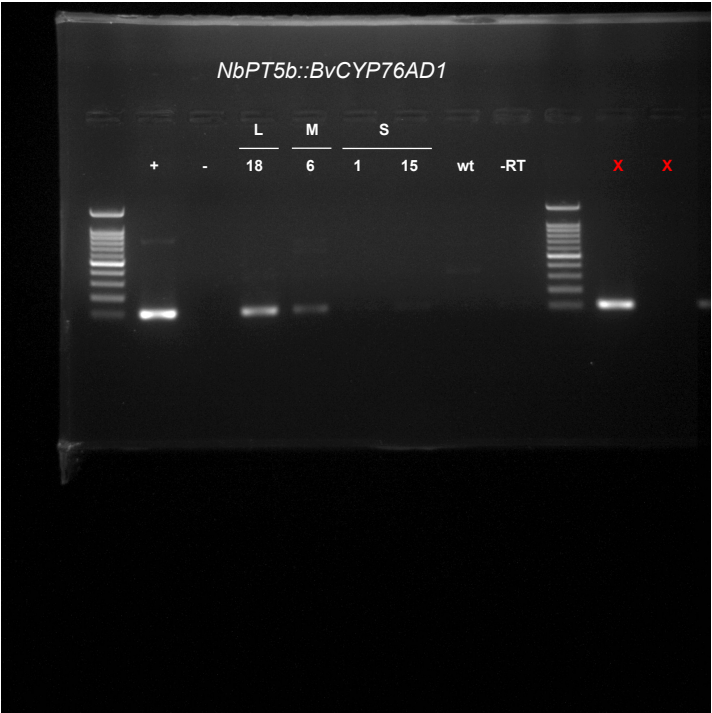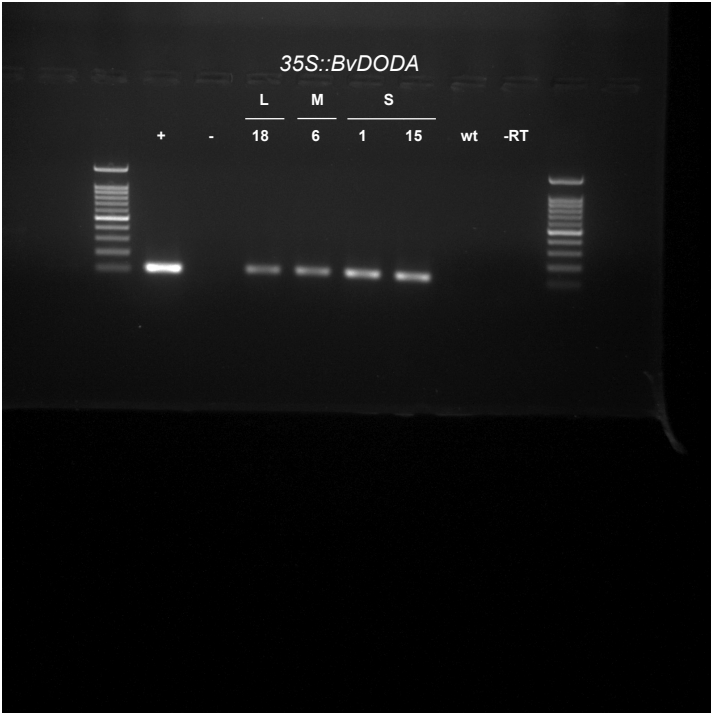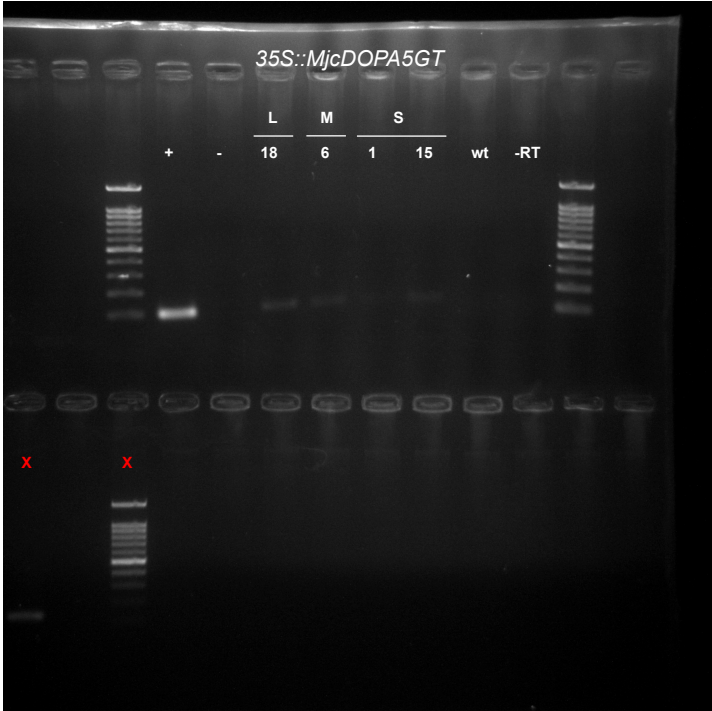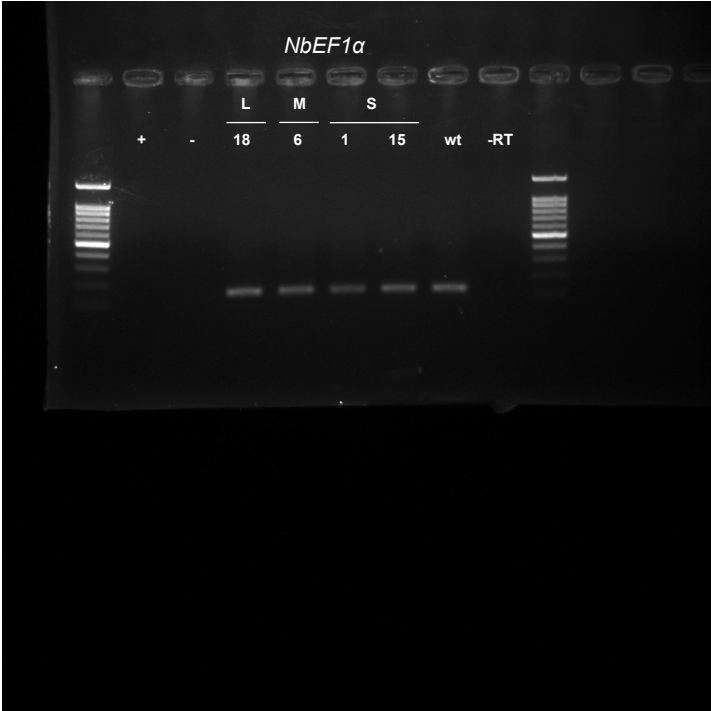

\* GD 100bp DNA Ladder RTU, (ready-to-use), DM001-R500, Newmarket Scientific

Supplement: S4 Data — (PDF) [file pbio.3001326.s022.pdf]
