# Supplementary material for: MycoRed: Betalain pigments enable in vivo real-time visualisation of arbuscular mycorrhizal colonisation
Source: PLoS Biol. 2021 Jul 14;19(7):e3001326. doi: 10.1371/journal.pbio.3001326 (PMC8312983; doi:10.1371/journal.pbio.3001326)
Supplement: Attachment — Submitted filename: REVISION_Response to reviewers.pdf [file pbio.3001326.s024.pdf]

RESPONSE TO REVIEWERS

MycoRed: Betalain pigments as in vivo real-time visual markers for arbuscular mycorrhizal colonisation of transgenic root systems

Timoneda A, Yunusov Y, Quan C, Gavrin A, Brockington SF, Schornack S

Editor's comments

The reviews of your manuscript are appended below. You will see that the reviewers find the work potentially interesting. However, based on their specific comments and following discussion with the academic editor, I regret that we cannot accept the current version of the manuscript for publication. We remain interested in your study and we would be willing to consider resubmission of a comprehensively revised version that thoroughly addresses all the reviewers' comments. We cannot make any decision about publication until we have seen the revised manuscript and your response to the reviewers' comments. Your revised manuscript would be sent for further evaluation by the reviewers.

As you will see, the majority of reviewers found your work to be interesting and useful to the field. However, all of them raised several important concerns that we would need to see experimentally addressed before considering the manuscript further for publication. Among other issues, the reviewers ask that you confirm that the symbiosis is functional,

Response: We have now carried out additional experimentation showing that the symbiosis genes *MtSTR* and *MtRAM2* are transcriptionally induced upon colonisation of betalain producing roots with AM fungi.

provide evidence that your system works without false positives when other microbes are present

Response: We performed root infections of the NbPT5b-p3 and BCP1b-p3 lines with *Phytophthora palmivora* and no visible red accumulation of red betalain pigment was observed. Furthermore, in all our colonisation experiments we use non-sterile sand substrate and non-sterile plants. There are other microbes present at all times, yet the color development is specific to the colonisation by AM fungi which have been supplied in addition. This is exemplified in the Medicago *dmi3* mutants which carry the reporters, were exposed to non-sterile substrate for weeks but did not show Betalain pigment accumulation (see Fig. S13).

and investigate whether there is a quantitative relationship between colonization and amount of pigment present.

Response: We never claimed that there is a quantitative relationship between colonisation and the amount of pigment present. Our method and resource is not aimed at quantifying colonisation based on the amount of pigment, it has been specifically established to report the spatial distribution over time. We believe that additional experimentation to correlate pigment amount with colonisation level is beyond the scope of this manuscript.

We would ask that you address reviewer #1's concerns about the usefulness of the resource in the text, preferably describing how you see this being used as, indeed, it would seem to be a difficult tool to use in a wide variety of plant genetic backgrounds.

Response: We apologize that reviewer 1 must have missed our previous lines clearly describing the limitations of the system and that it requires transformation (first submission, line 526). Please note that our comments on natural variation referred to the interaction rather than a variety of plant genetic backgrounds only. Our understanding on natural variation among AM fungal isolates and their root system colonisation dynamics is very limited and this resource will enable such studies. We have now amended the text to make these points more clear.

The reviewers would also like the work to include information on how you plan to handle distribution of the resource you have created (seeds and DNA constructs). We suggest using a resource such as the Arabidopsis Biological Resource Center (https://abrc.osu.edu/researchers) or similar for distribution to improve the accessibility of your resource to the scientific community.

Response: We have deposited all relevant plasmids at Addgene and have added a list of these with their descriptions and Addgene identifiers in Supplementary Table 3. Seed material for *N. benthamiana* MycoRed reporter lines (*NbPT5b-p3* and *NbBCP1b-p3*) are stored at the Sainsbury Laboratory Cambridge University dedicated storage facility in controlled environmental conditions following the International Day Room Standard of 15% relative humidity and a temperature of 15˚C (https://www.slcu.cam.ac.uk/facilities/seedstore). Seed material will be shipped to anyone requesting them. We plan to ship at least 15 seeds per request. N. benthamiana has small dry seeds and we have already bulked-up sufficient amounts for 50 requests. Phytosanitary certificates can be provided where needed. We have now included this information on storage and accessibility of vectors and seeds to our Material and Methods section.

We appreciate that these requests represent a great deal of extra work, and we are willing to relax our standard revision time to allow you six months to revise your manuscript. We expect to receive your revised manuscript within 6 months.

Reviewer's Responses to Questions

Reviewer #1:

This manuscript describes the development of betalains produced by transgenes under AM-specific promoters. The biggest constraint is that the construct will have to be introduced into every plant under examination. Whenever a different genetic background is needed, it must

be introduced. In most species, such an introduction is a tedious process either by transformation or genetic crosses. So it doesn't easily lend itself to dissecting the genetic mechanism. It only allows for analysis in the same genetic background. In this reviewer's opinion, having to transform a large construct before the AM dynamics can be analyzed hardly counts as "significant outperformance" or offers the "potential to address, for the first time, a pressing biological question".

The authors are correct in stating that "The most powerful application of our betalain-based AM reporter lies in its ability to non-invasively document colonisation in fully developed root systems over time".

Response: Thank you for agreeing with our claim.

They listed a number of possibilities. However, they failed to use their system to actually address any of these issues, and to demonstrate how they are pressing issues that cannot be addressed otherwise.

Response: The guidelines of Plos Biology do not require a method or resource to be applied to address a new biological question. As confirmed by the reviewer we listed a number of possibilities. The most important one is the non-invasive tracking of the dynamic colonisation of a root system over time depending on local or systemic changes in its environment. We (and reviewer 2 - see their comment III) are not aware of another published method which can achieve such tracking non-invasively. We would however appreciate the reviewers' insight to be able to discuss this in our manuscript.

Their claim that "A second important application is in the survey of induced or natural genetic variation that impacts on root system colonisation" is unconvincing. Not only one would have to introduce this reporter system into every line of a natural variation collection, transformation by itself will result in positional effect due to the randomness of transgene insertion site, or in their own words "varying degrees of transgene expression that could

impact reporter intensity and functionality", which applies to variations between stably transformed lines as well as between hairy roots.

Response: We understand the limitations pointed by the reviewer, and we already acknowledge these in our statement on line 525 of our initial submission after the mentioned claim: "*Here its use is only limited by the transformability of the species* [...]". We have edited this sentence to be more specific about the type of genetic variation we refer to. Natural variation in this context refers to variation in the interactions such as natural isolates of different AM fungi which may have different colonisation differences within a root system. These aspects can well be addressed with a single transgenic reporter line being a host to a range of AM fungal species and strains.

My second concern is whether betalains are a robust reporter at all. Does it have enough resolution? In Medicago truncatula, the resolution seems rather low. The images are much more encouraging in N. benthamiana. It is hard to conclude which species is the outlier.

Response: Resolution is dictated by the imaging technology rather than the reporter. Root architecture and thickness also significantly differs between Medicago and *N. benthamiana.* We have not carried out high resolution imaging with Medicago roots. We have done this with *N. benthamiana* roots, the main species of interest in this study. The system allows for cell level resolution and labs working with specific plant systems will have optimised their imaging setup.

Again, as the authors pointed out, the most promising application may be live imaging at the whole root system level. It is unclear why more conventional reporters, such as luciferase, would not suffice.

Response: To date there is no conventional published reporter to sequentially study AM fungal colonisation extent within the same root system over time. Luciferase would require the invasive sequential application of luciferin substrate at each imaging step as well as a

light-sensitive camera setup. Accessibility of luciferin to the inner root cortex cells colonised by AM fungi would not be homogenous. Furthermore, this would also be a transgenic reporter. I hope this reviewer agrees that a visual reporter which can be imaged using a simple flatbed scanner and without the application of expensive substrate allows for a wider range of research teams to utilise them easily.

Reviewer #2:

The manuscript by A. Timoneda et al. is a "method and resources" article that presents MycoRed, a method to visualize the occurrence of arbuscular mycorrhizal symbiosis in roots, in a non-destructive manner. The method is based on the expression of the betalain biosynthesis genes under control of AM symbiosis-specific promoters.

The authors tested the systems in two dicots, the legume Medicago truncatula and Nicotiana benthamiana. They isolated AM symbiosis-specific promoters in N. benthamiana. They identified and tested constructs that are not expressed when the plants are not inoculated, which is essential for further use of the system (low false-positive rate). Lastly, they used a rhizotron system to show the potential for real-time monitoring of AM symbiosis.

Symbiotic interactions are attracting more and more attention due to their potential as biofertilizer. This makes the development of such a tool particularly relevant and timely. As an expert in AM symbiosis research, I can foresee the potential for forward-genetic screens, which have been almost ignored due to technical difficulties, or to decipher cross interactions with biotic and abiotic factors. It is a much needed system and shows a great potential.

Response: Thank you for this very positive view on the potential of our resource.

I found the manuscript very clearly written and the reported experiments well conducted.

As this article is submitted as a resource to be widely used by the community, I have, however, a few additional requests and suggestions.

Major requests:

1. Confirm that the symbiosis is functional. Although the colonization of the transgenic lines, and the increased colonization rate observed over time, suggests that the association is "normal", it would be important to demonstrate it. As a proxy the authors should provide qRT-PCR data for the expression of marker genes such as RAM2 or STR/STR2 in the engineered lines following colonization by R. irregularis.

Response: As requested we have performed qRT-PCR for the MtSTR, MtRAM2 marker genes on *MtPT4*-p1 *Medicago truncatula* hairy roots and shown their expression is upregulated in roots which are colonised and have formed red betalain pigment (see revised Fig. S1). This supports the reviewers point that symbiosis is functional in the presence of betalains..

2. Demonstrate the specificity. The authors tested the lines in artificial substrate and in vitro. The presented data are very convincing, however I am wondering whether the lines would behave similarly in more natural, microbe-rich, soils. The authors should test for the potential activation of betalain production in the "elite" line (NbPT5b-p3) in presence of microbes other than AM fungi. Including a pathogen that infect the roots would be particularly relevant.

Response: We have carried out our AM colonisation experiments in non-sterile substrates supplemented with AM fungal spores. Figure S13 shows that in Medicago *dmi3* mutants which have been cultivated in non-sterile substrate for weeks, no betalain activation is observed, this suggests that the reporters cannot be activated by other substrate microbiota present. We have now carried out additional experimentation to address this aspect in *N. benthamiana* under controlled infection with a root pathogen. We have carried out root

infections of the *NbPT5b*-p3 and *NbBCP1b*-p3 lines with *Phytophthora palmivora*. No visible red accumulation of red betalain pigment was observed (see new Fig. S11). Furthermore, RNAseq data obtained previously show no evidence for expression of *NbPT5b* - Niben101Scf02726g00004.1 and *NbBCP1b* - Niben101Scf07438g04015 in published *P. palmivora N. benthamiana* root infection (Evangelisti *et al.*, 2017; https://doi.org/10.1186/s12915-017-0379-1, see supplementary material, both identifiers are absent from additional file 5 where all differentially expressed genes during a 72h infection time course have been reported). This supports our finding that the *N. benthamiana* promoters utilised in these lines are responsive to AM fungal colonisation in a similar fashion to the more widely studied *Medicago truncatula* homologs *MtBCP1* and *MtPT4*.

Suggestions:

1. The authors could provide a list (with reference numbers) for the L0 and L2 used for the study as well as a clear statement on the procedure to obtain these DNA modules, and .gb files of the vectors.

Response: We have deposited the L0 and L2 plasmids used in this manuscript in Addgene. A complete list of these and their Addgene reference numbers can now be found in new Supplementary Table 3.

2. The authors could explain the procedure to obtain seeds of the stable N. benthamiana NbPT5b-p3, including the amount that can be possibly provided. Again, this is a resource and distribution is an important aspect.

Response: *N. benthamiana* produces a significant amount of small dry seeds which can be easily harvested, stored and distributed. The authors can provide sufficient seeds to start their own plants to everyone requesting them (including phytosanitary certificates for shipments outside the UK where needed) and researchers can then grow their own stable

transgenic plants to harvest plenty more seeds. We have now included information on storage and accessibility of seeds to our Material and Methods section.

In addition the possibility to store seeds in the JIC Germplasm resource exists as well (https://www.jic.ac.uk/research-impact/germplasm-resource-unit/). We now state clearly in the manuscript that seeds are available.

3. I was particularly impressed by the rhizotron experiment, because following AM symbiosis in real-time is a challenge that has never been solved. I was wondering whether the authors could use the images to quantify a proxy for the level of colonization, on the color-extracted files (red / white for instance?). This is obviously not a requirement, but having a way to quantify colonization with a simple scan, on living material, would be amazing.

Response: Thank you for your positive words highlighting that our rhizotron experiment may allow us to solve the challenge following AM symbiosis in real-time. We agree with the reviewer developing a fast and easy protocol for color quantification as proxy for colonisation would be very useful. Efforts are already being conducted to develop appropriate imaging for this, but we unfortunately consider they fall outside the scope and time frame of this manuscript.

I am not an expert on the biosynthesis of Betalain and I did not evaluate that particular aspect of the manuscript.

Reviewer #3:

The manuscript by Timoneda and colleagues offers a nice technical contribution to the field of AM symbiosis. AM fungi develop inside the root tissues and there are only a few (and rare) morphological features that allow identifying the mycorrhizal root segments with the

naked eye. For this reason, the development of transgenic plants which allow an easy detection of the arbuscules is surely a useful contribution.

The research has been carefully performed with clear experiments and correct controls.

Response: Thank you for highlighting the usefulness of our contribution and your satisfaction with the way we carried out experiments.

There are however some points which deserve attention, and some missing information should be added: all these issues have been listed following the text.

Response: We have commented on your individual points below.

In addition, the text sounds as a bit redundant since the same experiments are described for Medicago and then for Nicotiana, even if the results and the conclusions are very similar. I fully understand that the stable transformation of Nicotiana was done in a second moment, but probably a shortening of the text could lead to a more attractive reading.

Response: Not the same, but similar experiments using different genetic resources as well as new promoters are described for Medicago and Nicotiana. We consider it important that the details of the experiments are laid out so that they are reproducible.

Title: is it clear that the visualisation of betalain pigments requires the introduction of reporter genes in model plants? In my opinion, the authors should state that a genetic transformation is at the basis of the experiment.

Response: We have renamed the title to '*Betalain pigments as* in vivo *real-time visual markers for arbuscular mycorrhizal colonisation of transgenic roots systems*'.

Summary line 20: probably the word fungi after arbuscular mycorrhiza is missing. AM are not fungi!

Response: Yes indeed. Thank you for spotting this. We have corrected it in the manuscript.

Introduction

Line 48. Glomeromycota. Please note that one of the most accepted taxonomy proposes AM fungi as Glomeromycotina, a subphylum (Spatafora et al 2016). Alternatively, see the taxonomic view as proposed by Leho Tedersoo Line 50 ".... can be formed by 70-90% of extant land plant species.". Please note that the references are not the most updated. It is assumed that only 70-72% of land plants are colonized by AM fungi (see Brundett and Tedersoo, 2018; Genre et al 2020); 90% is the percentage of plants which are associated to mycorrhizal fungi.

Response: Thank you for clarifying this, we have revised the text.

Line 57-60 Please note that also the other intracellular hyphae of AM fungi (those originated from the hyphopodia, the coils ..) are always surrounded by a host membrane. This new membrane is not exclusive of the arbuscules and is more correctly defined as the perifungal membrane.

Response: We have edited this introduction section and have removed the wording periarbuscular membrane.

78: "complex microscopy" not clear

Response: We have changed this to “light or fluorescence microscopy”

85-86 probably the yellow is not exclusive of cereals. Also Liliaceae have yellow mycorrhizal roots.

Response: Thank you for clarifying this, we have revised and updated the text.

96: Is there information on the natural expression of betalains in early diverging fungi?

Response: As mentioned in our manuscript (line 94-96), Betalains have only been found in the basidiomycetes Amanita and Hygrocybe. We are not aware of any other report or dataset on fungal betalain expression.

143--144 ".... Heterologous expression of betalain biosynthesis genes specifically driven by AM-responsive promoters effectively tracked AM colonisation dynamics in both species.." Perfect rationale, however, I would have appreciated to see the use of at least one gene also related to the first steps of colonization (i.e. signalling, transcription factors). These genes could give us more relevant information than the genes which are the markers of the established colonization

Response: For us the most relevant information on symbiosis is the expression of the phosphate transporter reporting the transfer of nutrients from fungus to plant. We agree that different research groups may have different preferences as to which aspect of colonisation they consider most relevant and there can be many different promoter-reporter fusions tested and generated but our method and resource focused on what in our opinion are two of the most relevant ones.

Line 163: Can the authors provide some further information on the UBQ10 promoter? And provide some rational for the use of the constitutive promoters, which indeed have not been the better choice.

Response: We have added a sentence further explaining the rationale behind this, i.e. to avoid transcriptional silencing due to repeated elements. The Ubi10 promoter was further described in the Methods section of our original submission, and we have now added its length in bp.

Fig. 2 it seems that the betalain red is mostly associated to the inner root segments and not to the thinnest root branches (lateral roots). Have the authors done some statistical evaluation of the staining distribution looking at the morphology of the root system?

Response: We established this reporter system to do exactly this in future research which is beyond the frame of this manuscript. At present we do not have sufficient data to conclude and correlation of root morphology and staining.

Fig. 3 The red seems to be more abundant in the endodermis cells, where surely PT4 is not expressed. The authors write that red betacyanin distribution extends to cells beyond those with promoter activity ...Can betacyanin move across membranes, given that it is water-soluble?

Response: Betalains are produced in the cytoplasm and stored in the vacuoles in native producing species. There is currently no information, however, on the transport mechanisms of betalain pigments. As small water-soluble compounds, betalains have the potential to move symplastically through root plasmodesmata, but this has not been proven. Nevertheless, expression in *N. benthamiana* roots seemed to be much more defined to single cells. It is also possible that these differences could be generated by slightly different sectioning methodologies. These points were covered in the discussion section of our original submission. We have now added a sentence acknowledging the possibility of betalain symplastic movement in the root.

172: can the betalain expression change the transcriptomic profile of arbuscule containing cells?

Response: We saw the formation of normal arbuscules. We have performed further experimentation to analyse expression levels of MtSTR and MtRAM2 and found they are normally induced in colonised Medicago lines producing red pigment. We have not tested whether other specific genes are altered in their expression as this would require a large scale experiment beyond the scope of this manuscript. However, we can conclude that any such potential changes did not impact on the formation of this symbiosis.

204-206. Medicago is a model plant for many labs. It is not clear why the authors moved to Nicotiana instead of producing stable Medicago transformants.

Response: Agreed, this could be done in several plants. We have opted for *N. benthamiana* as it is a model plant for many labs including in mycorrhiza symbiosis (doi: 10.1111/tpj.13908, Hause & Fester 2004 https://www.jstor.org/stable/23388865) and we are particularly interested in it. Furthermore it takes ~1.5 years to get stable homozygous Medicago. By contrast *N. benthamiana* takes 3-6 months to generate such a resource. In addition, *N. benthamiana* is more compatible with our rhizotron setup. Medicago roots are too thick and the root system is too big.

Fig. 4 Expression of NbPT5b and NbBCP1b increased after two weeks and showed significantly elevated transcript levels 3-4 weeks post inoculation (Fig 4a). In my opinion, this Fig 4 a does not show an original or unexpected result. I would suggest moving the Figure to the supplementary materials. Indeed the GUS constructs reveal a strong diffusion of the staining…

Response: We respectfully disagree. While the result might not be unexpected for the reviewer, the two *N. benthamiana* promoters have not been characterised previously, and solely concluding from sequence similarity of their downstream ORFs that they respond in a similar fashion to the better characterised Medicago promoters is not appropriate. We provide original and new data showing that two previously uncharacterised genes respond transcriptionally to AM fungal colonisation.

The two following paragraphs present many repetitions. Betalains can be used to visualise AM fungus colonisation in living Medicago truncatula roots vs Betalains visualise AM fungus colonisation in living Nicotiana benthamiana roots The authors should go in a more direct way to the solution, shortening the results obtained with the first constructs. It is clear that the constitutive promoters give problems...)

Response: We disagree that it is clear that the constitutive promoters give problems. Other published stable transgenic lines constitutively expressing betalain biosynthesis genes have not had reports of problems associated with them (Polturak et al., 2016, DOI: 10.1111/nph.13796; Polturak et al., 2017,https://doi.org/10.1073/pnas.1707176114). Therefore we consider it essential to report our observations.

Fig. 5 a "Schematic of the multi-gene vectors constructed for inducible betalain expression in N. benthamiana roots where only the first gene of the betalain biosynthesis pathway is controlled by AM symbiosis specific promoters". The legend is a bit confusing: the reader looks for differences between fig 5 vs. fig 2. But indeed the only difference is that promoters of CYP76AD1 are from Nicotiana and not from Medicago.

Response: Given the number of similar constructs used during this manuscript, we actively decided to include a schematic of each of them in their respective figures. We believe this will facilitate the reading process and a more effective acquisition of information. We regret that the reviewer does not agree with our style choice.

Fig 5 (e g) seems to be quite poor in quality.

Response: We consider the quality of the image sufficiently and effectively fulfills the objective of the figure which is to show fungal structure presence in those root regions. Internal hyphae, arbuscules and vesicles are clearly observable both in Fig 5e and 5g.

Table 1 the authors used ink staining to visualise the colonisation. But the method is not reported in the text (line 270) as well as the quantification methodology. Lastly, from which transformed plants were the data obtained?

Response: Data was obtained from N. benthamiana plants expressing NbPT5b-p1 and NbBCP1-p1 4 weeks post-inoculation with *R. irregularis* as stated in the text and the legend of Table 1 in lines 269 and 288 of our initial submission respectively. Methodology is also

summarised in line 269 of our initial submission and described in more detail in the“*Staining and quantification of fungal structures*” section of Material and Methods.

Paragraph "Stable expression of NbPT5b-p1 and NbBCP1b-p1 can cause shoot developmental defects in N. benthamiana". In my opinion this entire paragraph should be summarised, the details could be moved to the supplementary materials, since it is not so strictly relate to the main focus. On the other hand, it seems that the promoter PT5b is active in leaves. This could be an interesting result, even if not strictly related to this research. Is the transporter active in the leaves from Myc and not myc WT plants? It is known that PT is expressed also in root tips, irrespectively of the mycorrhizal colonization....

Response: All details on the developmental aspect are already exclusively described in supplementary figures. In an RNAseq experiment with N. benthamiana leaves exposed to control or pathogen infection (Carella et al, 2019 https://www.sciencedirect.com/science/article/pii/S096098221930692X, we did not obtain any reads for *NbPT5b* [Niben101Scf02726g00004.1] suggesting that this gene is not detectably expressed in leaves. We have discussed that Our *NbPT5b*-p1 construct transformation selection process could have been biased towards T0 plants with a degree of escaped *CYP76AD1* expression, which would explain the presence of betanin in a number of T1 plants descending from *CYP76AD1* shoot-expressing lines (Fig. S6, S8). Further experimentation is required to support this hypothesis, but in any case developmental defects and vegetative betanin expression can be avoided when all three biosynthesis enzymes are driven by AM symbiosis specific promoters.

Fig. 6 b-e do not add any relevant information.... it could be fused with Fig. 7. Fig. 8 is much more interesting.

Response: Figures 6 b-e add relevant information of the tissue and cell level distribution of pigment in colonised tissues. We consider this essential information in this manuscript.

In this context, it is not clear why the time course with the PT promoter is not shown, since it works better than the BCP one.

Response: Please note that in our original submission as well as in this version, both *NbBCP1b* and *NbPT5b* time course images were shown (see Fig. 8 for *NbBCP1b* and Fig. S15 for *NbPT5b*).

Line 348-350 - Vegetative? Not clear...do they mean shoot and leaves? Also roots are vegetative organs. And at line 350 "… vegetative betalain production.."

Response: Thank you for clarifying this, we have revised the text.

364 what is the meaning? Why do they come back to medicago? But.... does not make infection does not express PT4...since all the signalling is blocked and as a consequence the following processes…

Response: We have edited the manuscript for a more clear interpretation. *dmi3* plants were included in this transformation round as further negative controls, where we wouldn't expect any color production regardless of presence or absence of mycorrhizal inoculum. This also serves as proof that any other microbes present in our non-sterile substrate and which do not rely on the symbiosis signalling genes activate the reporter, a question which has been raised by other reviewers.

The last part of the discussion (from 451 line) should be deleted since the point has already been largely introduced and discussed in the Results

Response: We prefer to retain this short summary at the end of the manuscript, but are happy to remove it upon request by the editor.

Line 445: " A future solution to document total AM colonisation could involve the Establishment of systems whereby the betalain biosynthesis genes are activated by transactivators, which could be then driven by promoters that are active at early, main and

late AM fungal colonisation stages (44-46). I apologize, but for me the sentence is not clear. Transactivators?

Response: We wanted to highlight the possibility that establishing a betalain reporter line under control of an artificial promoter once will then allow crossing to many other lines which carry the transactivating transcription factor gene under control of one or multiple context specific promoters (e.g. promoter active at early and late stages of colonisation.

In conclusion, the authors should better show that the p3 constructs do not change plant and mycorrhizal phenotype, at least showing that the colonization percentage does not change between WT and transformants.

Response: We concur with the reviewer and have performed a new set of experiments to prove that the p3 constructs do not change colonization between WT and transformants. Specifically, we have colonised WT, pNbPT5b-p3 and pNbBCP1b-p3 transgenic *N. benthamiana* plants with *R. irregularis* over several weeks and then have stained and manually counted colonisation structures. No significant differences in root system colonisation as well as in the ratio of arbuscules in colonised sections were observed.

Reviewer #4:

The manuscript by Timoneda and coworkers presents the results of using Betalain biosynthetic genes to demonstrate effectiveness of the natural pigments as in vivo visual markers for arbuscular mycorrhizal colonization of both Medicago and tobacco root systems. They used AM-inducible promoters and assembled multi-gene reporter constructs and demonstrated that the innovative method MycoRed could allow for the non-invasive tracing of fungal colonization over time. The presented results fully support the conclusions and the study looks overall very convincing and solid. This research is of broad interest to the

audience of plant biologists and timely places among an increasing interest and importance of investigating AM.

Response: We thank the reviewer for their positive insight of our work.

The text reads fluently and the state of the art, results and conclusions are very clear. However, I have the following a few critics to raise.

Major points:

1. In this study, promoters of late AMS marker genes (MtPT4, MtBCP1, and tobacco homologs) were used to drive expression of betalain biosynthetic genes. The early infection and AM colonization is an essential part of AMS research, I would suggest testing the early AMS marker genes such as AM1 and AM3 and assay if the red pigmentation can be used to trace the infection at early stage of mycorrhizal infection, i.e., before 14 dpi.

Response: We agree with the reviewer that testing early AMS marker genes would be of great interest, but we unfortunately consider this falls out of the scope of our manuscript, which has been designed as a proof of concept of betalains as markers for colonisation. We chose two promoters that have been extensively studied and used in previous AMS research and which represent hyphal colonisation as well as arbuscule stages.

2. Stable transgenic tobacco plants were used to trace fungal colonization. A quantitative study could be performed to compare betalain pigmentation (by imaging or by HPLC) and fungal biomass accumulation (by qRT-PCR of R. irregularis housekeeping genes) and test if linear correlations exist.

Response: We also agree with the reviewer that a correlation of pigment intensity and colonisation levels could be of great use and we would really like to see this developed in the future. But we respectfully disagree with the experiment suggested, the purpose of this

paper was to show spatial correlation and we do not consider we need to perform such experimentation to support our conclusions.

Minors:

Figure 3: Panels c and d, overlay images of pigments (red) and WGA-FITC staining (blue) should be provided.

Response: We provide overlays for the reviewers, however we do not think that overlay images of these panels communicate the data in a more clear way. Therefore we prefer not to include it in the manuscript unless requested by the editor.

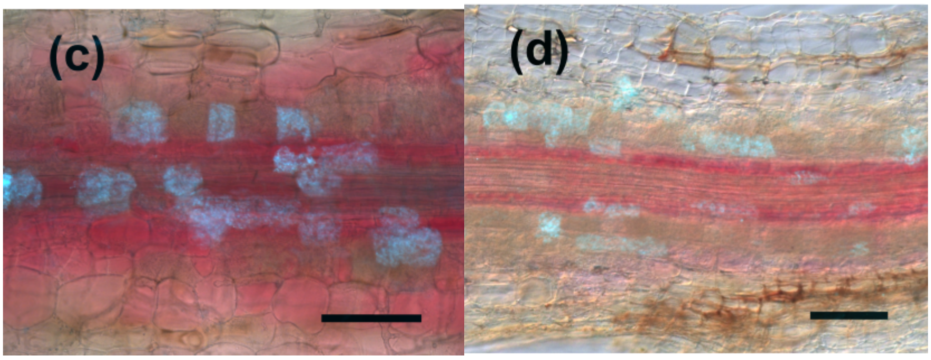

Figure 5: Overlay images of panels d&e, f&g are recommended.

Response: We here provide overlays as suggested by the reviewer. We believe this visualisation does not make the data clearer (especially for color vision impaired readers). Therefore we prefer not to include it in the manuscript unless requested by the editor.

Merged d&e) - *NbPt5b*-p1

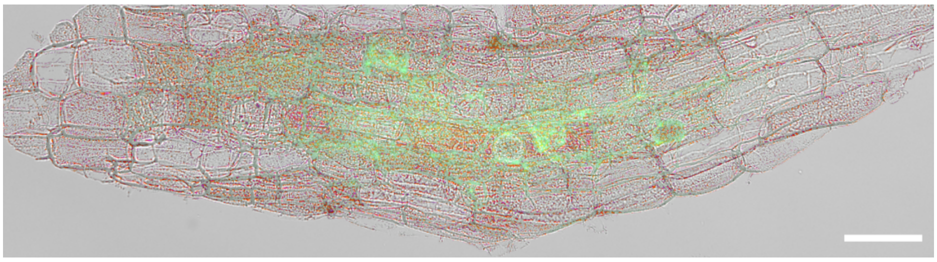

Merged f&g) - *NbBCP1b*-p1

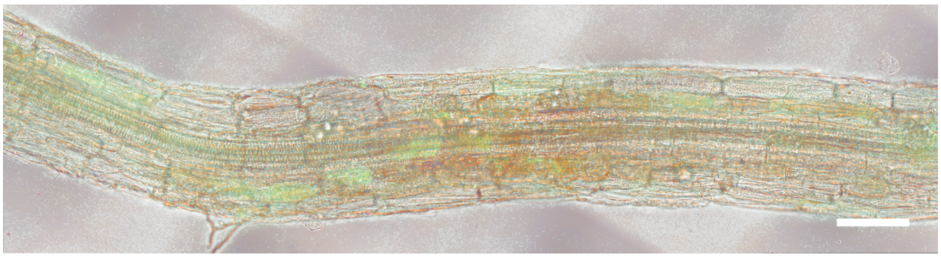

Figure 8: All dashed squares should be delimited in panel b.

Response: We initially designed the figure to include dashed squares in all panels, but we realised the repetitiveness of the squares were very obstructing for the proper visualisation of the figure. We hope that by indicating in the legend that this is "*is a magnification of the area delimited by the dashed square over time*" we make it more clear to the reader.

Figure S9: Panel j, what are the two types of arrows?

Response: Thank you for spotting this. We have corrected it in the manuscript.

Figure S11: Scale bars are missing.

Response: Thank you for spotting this. We have corrected it in the manuscript.

Table 1: 4.8±13? 3.0±8? 1.1±3?

Table S1: 8.8±17? 2.4±7?

Response: The error values shown in Table 1 and Table S1 represented the standard deviation of the data. We have now changed this to represent the standard error. We hope with this we align with the reviewer’s request.

Line 235: Definition of NbEF is needed.

Response: Thank you for clarifying this, we have revised and edited the text.

Line 263: d,f should be d,e

Response: Thank you for spotting this. We have corrected it in the manuscript.

Line 266: d-d should be d-g.

Response: Thank you for spotting this. We have corrected it in the manuscript.

Line 399: (a, b) should be (a, c)

Response: Thank you for spotting this. We have corrected it in the manuscript.

Lines 571-574: space is need between digits and units, i.e., 40 mM instead of 40mM.

Response: Thank you for clarifying this, we have revised the text.

Line 591&592: NbEF and RiEF are inaccurate. Gene IDs or RefSeq should be provided.

Response: Reference sequence identifiers for NbEF: Niben101Scf04639g06007.1, RiEF: XM_025321412.1, and RiBtub: XM_025314309.1 are now included the manuscript.
