# Supplementary material for: MycoRed: Betalain pigments enable in vivo real-time visualisation of arbuscular mycorrhizal colonisation
Source: PLoS Biol. 2021 Jul 14;19(7):e3001326. doi: 10.1371/journal.pbio.3001326 (PMC8312983; doi:10.1371/journal.pbio.3001326)
Supplement: Attachment — Submitted filename: Report Mycored Bonfante.doc [file pbio.3001326.s023.doc]

Mycored

I am sure to have already submitted the repost more than 10 dauys ago.

Please check

paola bonfante

Dear Editor

I have read with interest the manuscript *MycoRed: Betalain pigments as in vivo real-time visual markers for arbuscular mycorrhizal colonisation of root systems*: it is a nice technical contribution to the field of AM symbiosis. All the research has been developed in a correct way. The description is very (perhaps too much) detailed, with great attention to some "negative" results, while other information seems unfortunately to be missing.

I am not fully sure about the biological meaning, even if it is a technical note, but this is surely up to the Editor!

Best wishes

..

The manuscript by Timoneda and colleagues offers a nice technical contribution to the field of AM symbiosis. AM fungi develop inside the root tissues and there are only a few (and rare) morphological features that allow identifying the mycorrhizal root segments with the naked eye. For this reason, the development of transgenic plants which allow an easy detection of the arbuscules is surely a useful contribution.

The research has been carefully performed with clear experiments and correct controls. There are however some points which deserve attention, and some missing information should be added: all these issues have been listed following the text. In addition, the text sounds as a bit redundant since the same experiments are described for Medicago and then for Nicotiana, even if the results and the conclusions are very similar. I fully understand that the stable transformation of Nicotiana was done in a second moment, but probably a shortening of the text could lead to a more attractive reading.

**Title**: is it clear that the visualisation of betalain pigments requires the introduction of reporter genes in model plants? In my opinion, the authors should state that a genetic transformation is at the basis of the experiment.

**Summary** line 20: probably the word ***fungi*** after arbuscular mycorrhiza is missing. AM are not fungi!

**Introduction**

Line 48. Glomeromycota. Please note that one of the most accepted taxonomy proposes AM fungi as Glomeromycotina, a subphylum (Spatafora et al 2016). Alternatively, see the taxonomic view as proposed by Leho Tedersoo

Line 50 ".... can be formed by 70-90% of extant land plant species."

Please note that the references are not the most updated. It is assumed that only 70-72% of land plants are colonized by AM fungi (see Brundett and Tedersoo, 2018; Genre et al 2020); 90% is the percentage of plants which are associated to mycorrhizal fungi.

Line 57-60 Please note that also the other intracellular hyphae of AM fungi (those originated from the hyphopodia, the coils ..) are always surrounded by a host membrane. This new membrane is not exclusive of the arbuscules and is more correctly defined as the perifungal membrane.

78: "complex microscopy " not clear

85-86 probably the yellow is not exclusive of cereals. Also Liliaceae have yellow mycorrhizal roots.

96: Is there information on the natural expression of betalains in early diverging fungi?

143--144 ".... Heterologous expression of betalain biosynthesis genes specifically driven by AM-responsive promoters effectively tracked AM colonisation dynamics in both species*.."*

Perfect rationale, however, I would have appreciated to see the use of at least one gene also related to the first steps of colonization (i.e. signalling, transcription factors). These genes could give us more relevant information than the genes which are the markers of the established colonization

Line 163: Can the authors provide some further information on the UBQ10 promoter? And provide some rational for the use of the constitutive promoters, which indeed have not been the better choice.

Fig. 2 it seems that the betalain red is mostly associated to the inner root segments and not to the thinnest root branches (lateral roots). Have the authors done some statistical evaluation of the staining distribution looking at the morphology of the root system?

Fig. 3 The red seems to be more abundant in the endodermis cells, where surely PT4 is not expressed. The authors write that red betacyanin distribution extends to cells beyond those with promoter activity ...Can betacyanin move across membranes, given that it is water-soluble?

172: can the betalain expression change the transcriptomic profile of arbuscule containing cells?

204-206. Medicago is a model plant for many labs. It is not clear why the authors moved to Nicotiana instead of producing stable Medicago transformants.

*Fig. 4 Expression of NbPT5b and NbBCP1b increased after two weeks and showed  significantly elevated transcript levels 3-4 weeks post inoculation (Fig 4a). In my opinion, this* Fig 4 a does not show an original or unexpected result. I would suggest moving the Figure to the supplementary materials. Indeed the GUS constructs reveal a strong diffusion of the staining...

The two following paragraphs present many repetitions. *Betalains can be used to visualise AM fungus colonisation in living Medicago truncatula roots* vs *Betalains visualise AM fungus colonisation in living Nicotiana benthamiana roots* The authors should go in a more direct way to the solution, shortening the results obtained with the first constructs. It is clear that the constitutive promoters give problems... )

*Fig. 5 a "Schematic of the multi-gene vectors constructed for inducible betalain expression in N. benthamiana roots where only the first gene of the betalain biosynthesis pathway is controlled by AM symbiosis specific promoters"*

The legend is a bit confusing: the reader looks for differences between fig 5 vs. fig 2. But indeed the only difference is that promoters of CYP76AD1 are from Nicotiana and not from Medicago

Fig 5 (e g) seems to be quite poor in quality.

Table 1 the authors used ink staining to visualise the colonisation. But the method is not reported in the text (line 270) as well as the quantification methodology. Lastly, from which transformed plants were the data obtained?

Paragraph "*Stable expression of NbPT5b-p1 and NbBCP1b-p1 can cause shoot developmental defects in N. benthamiana "*

In my opinion this entire paragraph should be summarised, the details could be moved to the supplementary materials, since it is not so strictly relate to the main focus. On the other hand, it seems that the promoter PT5b is active in leaves. This could be an interesting result, even if not strictly related to this research. Is the transporter active in the leaves from Myc and not myc WT plants? It is known that PT is expressed also in root tips, irrespectively of the mycorrhizal colonization....

Fig. 6 b-e do not add any relevant information.... it could be fused with Fig. 7. Fig. 8 is much more interesting. In this context, it is not clear why the time course with the PT promoter is not shown, since it works better than the BCP one.

Line 348-350

Vegetative? Not clear...do they mean shoot and leaves? Also roots are vegetative organs. And at line 350 “… vegetative betalain production..”

364 what is the meaning? Why do they come back to medicago?

But.... does not make infection does not express PT4...since all the signalling is blocked and as a consequence the following processes...

The last part of the discussion (from 451 line) should be deleted since the point has already been largely introduced and discussed in the Results

Line 445: " A future solution to document total AM colonisation could involve the

Establishment of systems whereby the betalain biosynthesis genes are activated by transactivators, which could be then driven by promoters that are active at early, main and late

AM fungal colonisation stages (44–46).

I apologize, but for me the sentence is not clear. Transactivators?

In conclusion, the authors should better show that the p3 constructs do not change plant and mycorrhizal phenotype, at least showing that the colonization percentage does not change between WT and transformants.
