# Supplementary material for: MycoRed: Betalain pigments enable in vivo real-time visualisation of arbuscular mycorrhizal colonisation
Source: PLoS Biol. 2021 Jul 14;19(7):e3001326. doi: 10.1371/journal.pbio.3001326 (PMC8312983; doi:10.1371/journal.pbio.3001326)
Supplement: Attachment — Submitted filename: Response to reviewers.docx [file pbio.3001326.s025.docx]

**Response to editor and reviewer**

Thank you for submitting your revised Methods and Resources manuscript entitled "MycoRed: Betalain pigments as in vivo real-time visual markers for arbuscular mycorrhizal colonisation of transgenic root systems" for publication in PLOS Biology. I have now obtained advice from three of the original reviewers and have discussed their comments with the Academic Editor, who has also checked your responses to the missing reviewer.

Based on the reviews, we will probably accept this manuscript for publication, provided you satisfactorily address the data and other policy-related requests included below. In addition, we would like you to consider a suggestion to improve the title, which is quite long:

"MycoRed: a versatile method for in vivo real-time visualization of arbuscular mycorrhizal colonisation of root systems"

**Response:** We have modified the title of the manuscript to “MycoRed: Betalains enable *in vivo* real-time visualization of arbuscular mycorrhizal colonisation” which is 5 words shorter than the original.

As you address these items, please take this last chance to review your reference list to ensure that it is complete and correct. If you have cited papers that have been retracted, please include the rationale for doing so in the manuscript text, or remove these references and replace them with relevant current references. Any changes to the reference list should be mentioned in the cover letter that accompanies your revised manuscript.

**Response:** We have reviewed our reference list to ensure that it is complete and correct.

We expect to receive your revised manuscript within two weeks.

To submit your revision, please go to https://www.editorialmanager.com/pbiology/ and log in as an Author. Click the link labelled 'Submissions Needing Revision' to find your submission record. Your revised submission must include the following:

- a cover letter that should detail your responses to any editorial requests.

**Response:** We initially provided our responses in the cover letter, but were then requested by the Editorial Assistant to write a response to reviewers which we now supply.

------------------------------------------------------------------------

DATA POLICY: IMPORTANT - PLEASE READ

You may be aware of the PLOS Data Policy, which requires that all data be made available without restriction: http://journals.plos.org/plosbiology/s/data-availability. For more information, please also see this editorial: http://dx.doi.org/10.1371/journal.pbio.1001797

Note that we do not require all raw data. Rather, we ask that all individual quantitative observations that underlie the data summarized in the figures and results of your paper be made available in one of the following forms:

1) Supplementary files (e.g., excel). Please ensure that all data files are uploaded as 'Supporting Information' and are invariably referred to (in the manuscript, figure legends, and the Description field when uploading your files) using the following format verbatim: S1 Data, S2 Data, etc. Multiple panels of a single or even several figures can be included as multiple sheets in one excel file that is saved using exactly the following convention: S1_Data.xlsx (using an underscore).

2) Deposition in a publicly available repository. Please also provide the accession code or a reviewer link so that we may view your data before publication.

Regardless of the method selected, please ensure that you provide the individual numerical values that underlie the summary data displayed in the following figure panels as they are essential for readers to assess your analysis and to reproduce it:

Fig. 4A; Fig. S1; Fig. S8B and Fig. S10A, B

NOTE: the numerical data provided should include all replicates AND the way in which the plotted mean and errors were derived (it should not present only the mean/average values).

**Response:**
We have provided additional S3 Data which contains an excel sheet with all raw values to support the data displayed in Figure 4a, S1 Fig, S8b Fig, and S10 Fig. The numerical data provided includes all biological and technical replicates.

We have also updated qRT-PCR methods to further describe how the plotted data was calculated from raw values.

Please also ensure that figure legends in your manuscript include information on WHERE THE UNDERLYING DATA CAN BE FOUND, and ensure your supplemental data file/s has a legend.

**Response:** We have edited figure legends of Figure 4a, S1 Fig, S8b Fig, and S10 Fig to state raw quantitative values can be found in S3 Data.

Please ensure that your Data Statement in the submission system accurately describes where your data can be found.

**Response:** We stated in the submission system that all data can be found in the manuscript and supporting information.

-------------------------------------------------------------------------

BLOT AND GEL REPORTING REQUIREMENTS:

We require the original, uncropped and minimally adjusted images supporting all blot and gel results reported in an article's figures or Supporting Information files. We will require these files before a manuscript can be accepted so please prepare and upload them now. Please carefully read our guidelines for how to prepare and upload this data: https://journals.plos.org/plosbiology/s/figures#loc-blot-and-gel-reporting-requirements

**Response:** We provide a single PDF file that contains all the original uncropped and unedited gel images contained in S6 Fig. The file is named ‘S4_Data’ is also included as a supplementary file.

----------------------------------------------

Reviewers’ comments

Rev. 2: Pierre-Marc Delaux - note that this reviewer has signed his review.

In this revised version of their manuscript, the authors followed my three main suggestions:

1) They show convincing evidence that the expression of the betalain reporter does not impact symbiosis functionality. Although they only included data for Medicago it is reasonable to think that the same will be true for the Nb lines.

2) They provide additional evidence indicating the specificity of the reported (besides the fact that the substrate was not sterilized and thus contains other microorganisms).

3) They provide a detailled procedure to obtain the material, an essential aspect of this work submitted as a "Methods and Resources".

The reported conclusions are supported by the presented data and the work will be definitely an extremely useful resource for the plant symbiosis community. I do not have additional comments.

**Response:** Thank you for your positive review, and your comments on an earlier version.

Rev. 3:

The Authors strongly improved their manuscript and solved some of the major criticisms. Even if not all the questions were faced, the rebuttal letter was fully convincing.

**Response:** Thank you for supporting our manuscript and your comments on an earlier version.

Rev. 4: Deqiang Duanmu - note that this reviewer has signed his review.

Accept.

**Response:** Thank you for your and your comments on an earlier version.
